# Transport-coupled ubiquitination of the borate transporter BOR1 for its boron-dependent degradation

**DOI:** 10.1093/plcell/koaa020

**Published:** 2020-12-03

**Authors:** Akira Yoshinari, Takuya Hosokawa, Marcel Pascal Beier, Keishi Oshima, Yuka Ogino, Chiaki Hori, Taichi E Takasuka, Yoichiro Fukao, Toru Fujiwara, Junpei Takano

**Affiliations:** 1 Graduate School of Life and Environmental Sciences, Osaka Prefecture University, Sakai, 599-8531, Japan; 2 Graduate School of Agriculture, Hokkaido University, Sapporo, 060-8589 Hokkaido, Japan; 3 Institute of Transformative Bio-Molecules (WPI-ITbM), Nagoya University, Nagoya, 464-8601 Japan; 4 Graduate School of Agricultural and Life Sciences, the University of Tokyo, Tokyo 113-8657, Japan; 5 Plant Global Education Project, Nara Institute of Science and Technology, 8916-5 Takayama, Ikoma, 630-0101, Japan; 6 Department of Bioinformatics, Ritsumeikan University, 1-1-1, Nodihigashi, Kusatsu, 525-8577, Japan

## Abstract

Plants take up and translocate nutrients through transporters. In *Arabidopsis thaliana*, the borate exporter BOR1 acts as a key transporter under boron (B) limitation in the soil. Upon sufficient-B supply, BOR1 undergoes ubiquitination and is transported to the vacuole for degradation, to avoid overaccumulation of B. However, the mechanisms underlying B-sensing and ubiquitination of BOR1 are unknown. In this study, we confirmed the lysine-590 residue in the C-terminal cytosolic region of BOR1 as the direct ubiquitination site and showed that BOR1 undergoes K63-linked polyubiquitination. A forward genetic screen identified that amino acid residues located in vicinity of the substrate-binding pocket of BOR1 are essential for the vacuolar sorting. BOR1 variants that lack B-transport activity showed a significant reduction of polyubiquitination and subsequent vacuolar sorting. Coexpression of wild-type (WT) and a transport-defective variant of BOR1 in the same cells showed degradation of the WT but not the variant upon sufficient-B supply. These findings suggest that polyubiquitination of BOR1 relies on its conformational transition during the transport cycle. We propose a model in which BOR1, as a B transceptor, directly senses the B concentration and promotes its own polyubiquitination and vacuolar sorting for quick and precise maintenance of B homeostasis.

## Teaser

The borate transporter BOR1 senses the boron concentration through its borate transport activity for K63-linked polyubiquitination and subsequent degradation.

## Introduction

Plants take up and translocate mineral nutrients via roots using specific transporters. To deal with the fluctuating nutrient availability in the soil environment, plants tightly regulate the abundance of transporters by sensing nutrient concentrations. When a nutrient is limited, plant roots express a set of transporters for efficient transport. In this state, resupply of the nutrient could potentially cause rapid accumulation to a toxic level in plant tissues. To avoid this problem, several nutrient transporters in plants are downregulated by degradation through ubiquitination in response to elevated nutrient concentrations (Barberon et al., 2011; Bayle et al., 2011; Kasai et al., 2011; Lin et al., 2013; Rodriguez-Furlan et al., 2019).

Boron (B) is essential for plant growth and survival due to its function in cross-liking pectin at the rhamnogalacturonan II (RG-II; Yoshinari and Takano, 2017). Boron is taken up mainly as boric acid (B[OH]_3_) by plant roots from the soil solution by passive diffusion and channels (Funakawa and Miwa, 2015; Yoshinari and Takano, 2017). Under low-B conditions, a boric acid channel NIP5;1 and the borate anion [B(OH)_4_^–^] exporter BOR1 play key roles in B uptake and translocation in roots of *Arabidopsis thaliana* (Takano et al., 2002; Takano, 2006; Takano et al., 2010; Miwa et al., 2013; Yoshinari and Takano, 2017). In various root cells, including epidermal and endodermal cells, NIP5;1 and BOR1 are localized to the plasma membrane in a polar fashion toward the soil- and stele-side, respectively (Takano et al., 2010; Yoshinari and Takano, 2017). The polar localization of NIP5;1 and BOR1 supports directional transport of B toward the root stele (Wang et al., 2017; Yoshinari et al., 2019). BOR2, one of the BOR1 homologs in *Arabidopsis thaliana*, is similarly a B exporter for B translocation in the roots, and it is also involved in cross-linking of the RG-II to support root cell elongation under low-B conditions (Miwa et al., 2013).

Similar to most essential nutrients, excessive supply of B inhibits plant growth (Aibara et al., 2018). B toxicity affects various cellular activities, and B accumulation often causes necrosis of tissues (Landi et al., 2019). Since B accumulates at the end of the transpiration stream, high-B tolerance is related to the reduced accumulation of B in the shoot tissue, at least in wheat (*Triticum aestivum*), barley (*Hordeum vulgare*), and Arabidopsis (Nable, 1988; Nable et al., 1990; Reid et al., 2004; Reid, 2007). In Arabidopsis and barley, B exclusion from roots by borate exporters BOR4 and Bot1, respectively, protects plants from B accumulation and subsequent toxicity (Miwa et al., 2007; Sutton et al., 2007). In Arabidopsis, transport activity of B toward shoots is upregulated under low-B conditions and is rapidly downregulated after high-B resupply (Takano et al., 2005; Takano, 2006). This regulation is thought to be dependent on the abundance of NIP5;1 and BOR1. The abundance of the NIP5;1 protein is controlled through B-induced mRNA degradation dependent on the 5′ untranslated region (5′UTR; Tanaka et al., 2011, 2016). By contrast, abundance of the BOR1 protein is controlled by two different mechanisms. High concentrations of B repress the translation of BOR1 dependent on the 5′UTR (Aibara et al., 2018). In addition, high concentrations of B rapidly induce the ubiquitination of BOR1 followed by vacuolar transport and degradation (Takano et al., 2005; Kasai et al., 2011). Importantly, transgenic Arabidopsis plants expressing a ubiquitination-defective BOR1 variant (K590A) accumulated higher concentrations of B in shoots and exhibited a significant reduction of shoot growth under high-B conditions (Aibara et al., 2018). Plants expressing BOR1 lacking both endocytic degradation and translational repression exhibited further accumulation of B and a reduction of shoot growth. These results demonstrated the physiological importance of both mechanisms of BOR1 downregulation under toxic-B conditions. It should be noted that the translational repression occurs at higher B concentrations than the degradation of BOR1 protein (Aibara et al., 2018). Under continuous toxic-B conditions, stopping the de novo protein synthesis could be more cost-effective to control the BOR1 abundance. However, when the B concentration in soil fluctuates between low and sufficient ranges, the turnover of existing BOR1 protein should be particularly important because BOR1 is a key factor that determines the concentration of B in the stele for efficient root-to-shoot translocation under low-B conditions (Takano et al., 2002).

Subcellular localization and abundance of BOR1 are controlled by membrane trafficking. BOR1 in the plasma membrane is constantly endocytosed toward the *trans*-Golgi network/early endosome (TGN/EE; Viotti et al., 2010; Yoshinari et al., 2018). Under low-B conditions, the constitutive endocytosis is dependent on the AP2 clathrin-adaptor protein complex and DYNAMIN-RELATED PROTEIN 1A (DRP1A), and subsequent recycling maintains polar localization in the plasma membrane (Yoshinari et al., 2016, 2019). Upon high-B supply, however, BOR1 is further transported from the TGN/EE to luminal vesicles of the multivesicular body/late endosome (MVB/LE) and then to the vacuole for degradation (Takano et al., 2005; Viotti et al., 2010; Yoshinari et al., 2018). Interestingly, the B-induced degradation is dependent on DRP1A but not on the AP2 complex (Yoshinari et al., 2016, 2019), indicating differential routes of BOR1 endocytosis from the plasma membrane. Upon high-B supply, BOR1 is ubiquitinated presumably at a lysine residue (K590) in the C-terminal cytosolic tail (Kasai et al., 2011). It appears that ubiquitination triggers AP2-independent endocytosis specialized for vacuolar transport. However, the machinery of B sensing that promotes ubiquitination of BOR1 has not been elucidated.

In the present study, we analyzed the ubiquitination status of BOR1 upon high-B supply in depth and demonstrated that BOR1 undergoes K63-linked polyubiquitination at the K590 residue. We performed a forward genetic screen to identify a putative B-sensor/receptor and unexpectedly found that amino acid residues located in the substrate-binding pocket of BOR1 are essential for ubiquitination of BOR1. We propose a model in which BOR1 acts as a B-transceptor regulating vacuolar transport of itself.

## Results

### Polyubiquitination at K590 promotes vacuolar transport of BOR1

**Figure 1:**
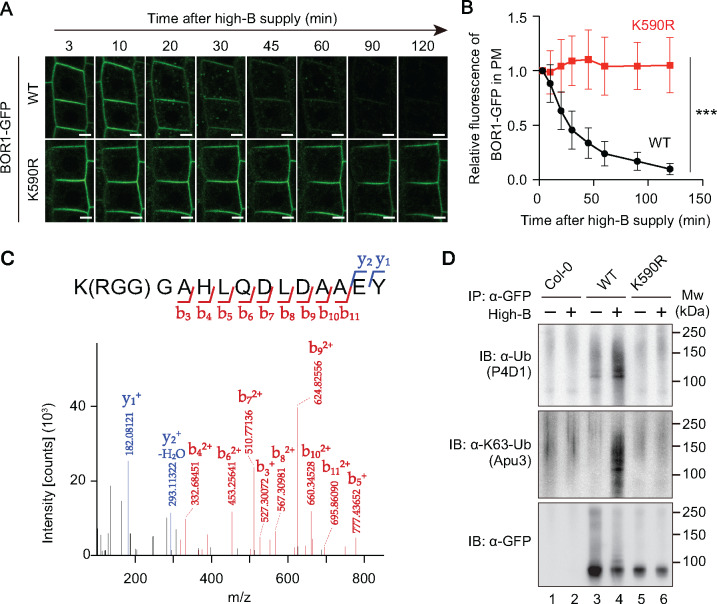
BOR1 undergoes polyubiquitination under high-B conditions. (A and B) Boron-induced vacuolar transport of BOR1-GFP was impaired by the K590R mutation. The 4-day-old seedlings grown with 0.5 µM B were shifted to high-B (100 µM) media. A, Confocal microscopy of WT and the K590R mutant of BOR1-GFP in root epidermal cells. Scale bars indicate 5 µm. B, Relative fluorescence of BOR1-GFP in the plasma membrane after high-B supply. Error bars represent mean ± SD. ***P <* *0.0001, determined by two-way ANOVA. *n = *67 (WT) and 68 (K590R) cells from three different roots. (C) BOR1 is directly ubiquitinated at the K590 residue. A representative result of tandem mass spectrometry (MS/MS) of a peptide derived from BOR1-GFP digested by chymotrypsin. A peak shift due to conjugation of Arg-Gly-Gly (RGG) to the K590 residue was detected. Protein was extracted from *pro35S:BOR1-GFP*/Col-0 plants grown with 0.5 µM B for 22 days. (D) Lysine-590 is required for polyubiquitination of BOR1. Immunoblot analysis of ubiquitination against BOR1-GFP. BOR1-GFP was immunoprecipitated using an anti-GFP antibody and then detected by anti-ubiquitin antibody (P4D1), anti-K63-linked polyubiquitination antibody (Apu3), and anti-GFP antibody, respectively.

Our previous report suggested that endocytic degradation of BOR1 is mediated by mono- or diubiquitination under high-B conditions (Kasai et al., 2011). A BOR1 variant with a lysine-to-alanine substitution at the 590th amino acid (K590A) is not ubiquitinated under high-B conditions, indicating that the K590 residue is essential for high B-induced ubiquitination (Kasai et al., 2011). However, no evidence of direct ubiquitination of the K590 has been shown. Hence, we initially attempted to investigate whether the K590 is ubiquitinated in response to a high-B treatment. To rule out the possibility that the electric charge alteration by the K590A mutation caused an abnormal conformation change of BOR1, we tested the vacuolar transport and ubiquitination of a BOR1 variant (K590R), which possesses the same positive charge as lysine. Similar to the previous results with K590A, time-lapse imaging of root epidermal cells after high-B supply (100 µM boric acid) showed that BOR1(K590R)-GFP remained in the plasma membrane for 120 min, while wild-type (WT) BOR1-GFP was rapidly internalized and degraded (Figure 1A and B). This result eliminates the possibility that the observed change of trafficking in the K590A mutant is due to the uncharged alanine.

Next, to examine whether the K590 residue is directly ubiquitinated, we performed liquid chromatography-tandem mass spectrometry (LC-MS/MS) of chymotrypsin-digested peptides of BOR1-GFP. The total protein was extracted from transgenic plants harboring *pro35S:BOR1-GFP* and the BOR1-GFP protein was enriched by immunoprecipitation (IP) using an anti-GFP antibody (Supplemental Figure 1). The footprint of ubiquitin (RGG, Arg-Gly-Gly) was detected at the K590 residue of the peptide fragment [^590^K(RGG)GAHLQDLDAAEY] (Figure 1C;Supplemental Figure 1B).

To analyze the ubiquitination status of WT and K590R-substituted BOR1-GFP, we conducted IP of BOR1-GFP and detected ubiquitin-conjugated proteins using anti-ubiquitin antibodies (Figure 1D;Supplemental Figure 2 and 3). Immunoblotting with an antibody against ubiquitin, P4D1, showed ladder-like signals from ∼100 kDa to ∼200 kDa above the position of WT BOR1-GFP (98 kDa) detected by an anti-GFP antibody. The signals detected by the P4D1 antibody in lanes with WT BOR1-GFP increased in response to high-B treatment (Figure 1D, Lane 4; Supplemental Figures 2 and 3) and did not appear in the corresponding lanes with Col-0 control and BOR1(K590R)-GFP (Figure 1D, Lane 1, 2, 5, and 6, respectively; Supplemental Figures 2 and 3). Importantly, immunoblotting with a chain type-specific antibody against K63-linked ubiquitination, Apu3, also showed the ladder-like signals above the position of WT BOR1-GFP in the sample treated with high-B (Figure 1D, Lane 4). We also tested whether BOR1 undergoes K48-linked ubiquitination, which is known to act in proteasome-mediated proteolysis, using a chain type-specific antibody Apu2. However, we did not detect any positive signals, which would have been indicative of K48-linked ubiquitination of BOR1 (Supplemental Figure 3). These data suggest that BOR1 undergoes K63-linked polyubiquitination in response to high-B treatments. We conclude that BOR1 undergoes ubiquitination at the K590 residue and that high-B concentrations induce K63-linked polyubiquitination.

### Genetic screening identifies amino acid residues within BOR1 required for vacuolar transport

**Figure 2:**
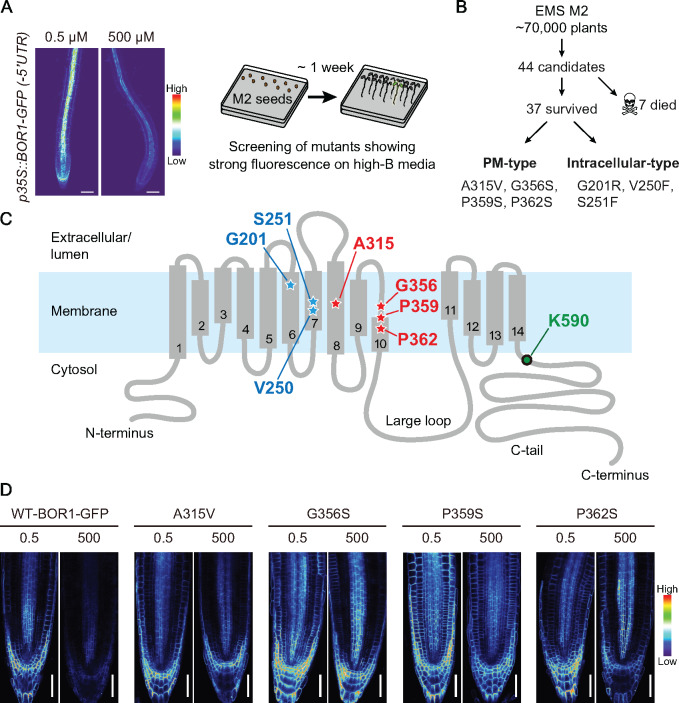
Forward genetic screen identified internal mutations that affect vacuolar transport and subcellular localization of BOR1-GFP. (A) Experimental design of the mutant screening. Representative images of BOR1-GFP driven by *35S* promoter under low (0.5 µM) and high (500 µM) B conditions, which were used for the screening (left). Confocal microscopy of *pro35S:BOR1-GFP*/Col-0 plants grown on medium containing 0.5 or 500 µM B for 5 days. Scale bars represent 50 µm. Conceptual illustration of the forward genetic screen to identify mutants in which vacuolar transport of BOR1-GFP is impaired (right). M2 plants exhibiting strong fluorescence of BOR1-GFP on the high-B (500 µM B) media were selected. (B) Brief summary of mutant screening. (C) Topological model of BOR1 protein. Amino acid residues identified as missense mutations in the screening and the ubiquitination site (K590) are highlighted. (D) Fluorescence of BOR1-GFP variants in M4 seedlings grown on 0.5 or 500 µM B for 5 days. Scale bars represent 50 µm.

To identify the “boron sensor/receptor” that controls BOR1 ubiquitination in response to the elevation of boric acid concentrations, we performed a forward genetic screen using an *Arabidopsis thaliana* transgenic line harboring *pro35S:BOR1-GFP(-5′UTR)*, which constitutively expresses BOR1-GFP without translational repression under high-B conditions (Aibara et al., 2018). When plants were grown on high-B medium (500 µM boric acid), BOR1-GFP was degraded, and thus showed weaker fluorescence than on low-B (0.5 µM) medium (Figure 2A). We screened for mutants in an ethyl-methane sulfonate (EMS) treated M2 population showing strong fluorescence of BOR1-GFP under the high-B condition (Figure 2A). Through the screen, we isolated 44 candidates showing a bright fluorescence of BOR1-GFP under high-B conditions from the pool of 70,000 M2 seedlings (Figure 2B).

Among the 44 candidates, 7 showed severe developmental defects and did not produce progenies. We then sequenced the BOR1-GFP transgene in the 37 candidates and identified 7 missense mutations within the protein-coding sequence. These BOR1 variants can be separated into two groups in terms of their subcellular localization, a plasma membrane localization-type (PM-type; A315V, G356S, P359S, and P362S) and an intracellular-localization type (G201R, V250F, and S251F). V250F and S251F were isolated repeatedly from distinct M2 pools, indicating that the screen reached near saturation. G201 is located in the transmembrane domain (TMD) 6, V250, and S251 are located in the TMD 7, A315 is located in the TMD 8, and G356, P358, and P362 are located in the TMD 10 or its vicinity (Figure 2C). It should be noted that the K590 residue, which is encoded as AAA, is theoretically not affected by EMS, which frequently produces G to A (C to T) base pair changes through *N-*7 alkynylation of guanine rather than other nucleotides (Sega, 1984).

In contrast to WT, the intracellular-type BOR1-GFP variants colocalized with intracellular compartments stained by ER-Tracker Red in the lateral root cap cells (Supplemental Figure 4A) and showed a network pattern in cotyledon epidermal cells (Supplemental Figure 4B). These results suggest that G201R, V250F, and S251F substitutions abolished proper folding of BOR1-GFP and consequently disturbed the exit of BOR1-GFP from the endoplasmic reticulum (ER; Ellgaard and Helenius, 2003). Furthermore, these variants were not degraded under a high-B condition (Supplemental Figure 4C), indicating that initiation of B-induced vacuolar transport of BOR1 does not take place at the ER membrane or requires properly folded BOR1 protein.

Next, to characterize the PM-type BOR1-GFP variants, we grew the mutant seedlings under two different B concentrations and observed their subcellular localization and fluorescence intensity in primary root tips (Figure 2D). WT BOR1-GFP showed a decrease of the fluorescence intensity under a high-B condition (500 µM boric acid; Figure 2D). WT BOR1-GFP was faintly observed under a high-B condition, while the A315V, G356S, P359S, and P362S mutants still showed a clear localization in the plasma membrane (Figure 2D). Intriguingly, all of the substitutions are not in the proximity of the K590 ubiquitination site but located in the TMDs.

### Substitutions of amino acids located within the substrate-binding pocket of BOR1-affected degradation and B-transport activity

**Figure 3:**
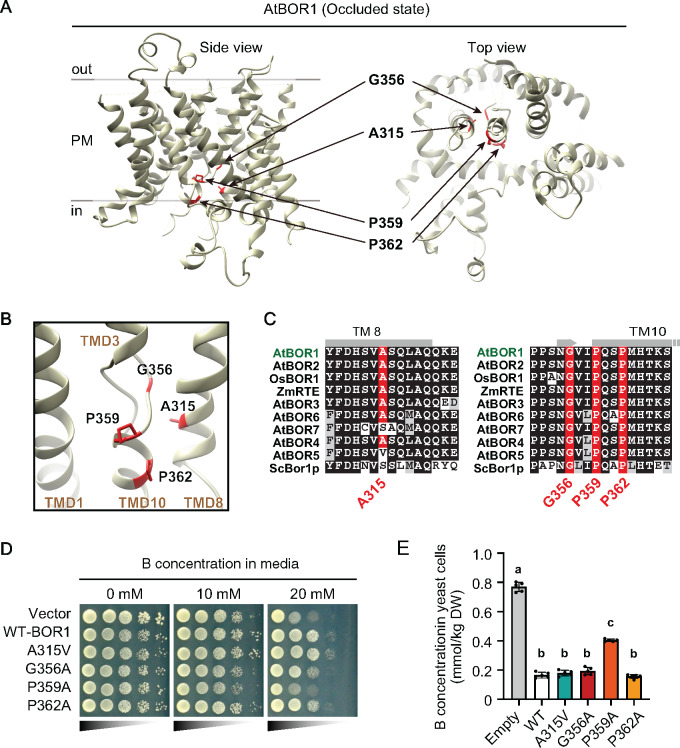
Amino acid residues A315, G356, P359, and P362 surround the potential substrate-binding site. (A) Three-dimensional structure of *Arabidopsis thaliana* BOR1 in the occluded state (PDB 5L25). (B) Substrate-binding pocket of BOR1. Structural images were depicted by UCSF Chimera software ver. 1.13.1 (Pettersen et al., 2004). (C) Multiple alignment of BOR1 orthologs in plants and budding yeast. (D and E) Transport activity of the BOR1 variants. (D) *S. cerevisiae bor1Δ* strains harboring AtBOR1 variants (WT, A315V, G356A, P359A, and P362A) were spotted on SD media containing 0, 10, or 20 mM boric acid and cultured for 6 days at 30°C. Diluted yeast suspensions were spotted onto the media with OD_600_=0.4, 0.04, 0.004, or 0.0004. (E) ^11^B content in the yeast cells after incubation with liquid medium containing 1 mM of boric acid for 50 min at 30°C. Different letters indicate significant differences between constructs, determined by one-way ANOVA followed by Tukey–Kramer’s post hoc test (*P *<* *0.05). *n *=* *4 or 5 different colonies were used. Error bars represent mean ± SD.

Next, we investigated the roles of the amino acid residues A315, G356, P359, and P362 in the B-transport function. These four amino acid residues are located in the putative substrate-binding pocket of BOR1, as indicated in a three-dimensional model reported in previous studies (Figures 3A and 3B; Coudray et al., 2016; Thurtle-Schmidt and Stroud, 2016). A multiple alignment indicated that G356, P359, and P362 are highly conserved in BOR1 homologs in plants, protists, and fungi (Figure 3C;Supplemental Figure 5). By contrast, A315 is conserved in clade I-BORs including BOR1 orthologs, but is less conserved in clade II-BORs including AtBOR4, which functions in B exclusion at high-B conditions (Miwa et al., 2007; Wakuta et al., 2015), and it is not conserved in the homologs in mosses (clade III-BORs), protists, and fungi (Figure 3C;Supplemental Figure 5; Supplemental Data set 1; Supplemental File 1). These substitutions in the vicinity of the substrate-binding pocket raise the question whether the degradation defect of PM-type variants is linked to the transport activity of BOR1.

To examine the involvement of these residues in borate transport activity, we introduced a series of BOR1 variants (A315V, G356A, P359A, and P362A) fused with Xpress-tag into the *bor1⊿* yeast (*Saccharomyces cerevisiae*) strain, that has a significantly reduced B-export capacity (Takano et al., 2007). In this and following experiments, we substituted G356, P359, and P362 to A to keep the nonpolar nature of the amino acids, although the substitutions we identified in the screening were S substitutions. The expression of functional BOR1 confers viability to yeast cells under toxic-B conditions by reducing B-concentrations in the cells (Takano et al., 2007). In yeast, the Xpress-tagged AtBOR1 was apparently not degraded under high-B conditions (Supplemental Figure 6A). Immunoblotting and immunostaining against the Xpress-tag showed comparable expression levels and PM localization of the BOR1 variants in yeast cells (Supplemental Figures 6B–D). Using these transformants, we tested whether the BOR1 variants could confer the tolerance to toxic-B levels in the media (Figure 3D). Introduction of WT AtBOR1 enhanced the yeast viability in comparison to the empty vector at 10 and 20 mM B supply (Figure 3D). Introduction of the A315V and P362A variants enhanced the viability at levels comparable to WT, while G356A and P359A showed markedly less viability (Figure 3D). A quantification of the intracellular B concentration in yeast transformants after supply of 1 mM boric acid revealed a significantly higher B concentration for the P359A variant compared to WT BOR1 [Figure 3E, *P *<* *0.001 by one-way analysis of variance (ANOVA) followed by Tukey–Kramer’s post hoc test]. By contrast, there was no significant difference between the B concentration in cells with the A315V, G356A, and P362A variants from the cells with WT BOR1, although G356A tended to show slightly higher values (Figure 3E). Taken together, the G356A substitution little affected the B-export activity, while the P359A significantly inhibited the B-export activity of BOR1 in the yeast cells. The A315V and P362A mutations, however, did not have an obvious effect on the B-transport activity of BOR1.

### The putative proton-binding site D311 is essential for B-transport activity of BOR1

Our results that missense mutations in the vicinity of the BOR1 substrate-binding pocket affected its B-induced degradation is reminiscent of the mechanisms underlying substrate-induced degradation of nutrient transporters in yeast (Babst, 2020). Recent studies on amino acid transporters Mup1, Can1, Gap1, and uracil transporter Fur4 indicated that substrate binding to the transport site induces a conformation transition and promotes their ubiquitination (Guiney et al., 2016; Gournas et al., 2017). Based on this regulatory model of yeast transporters, we hypothesized that substrate binding and conformational transition of BOR1 are involved in B-induced ubiquitination of K590.

**Figure 4:**
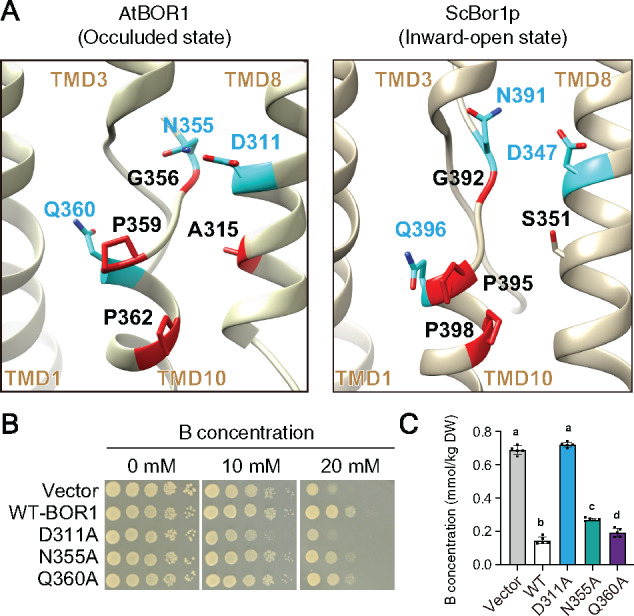
Boron-transport activity of BOR1 variants in yeasts. (A) Comparison of amino acid positions between AtBOR1 (PDB: 5L25) and ScBor1p (PDB: 5SV9). D311, N355, and Q360 in AtBOR1 are homologous to D347 and Q396 in ScBor1p, respectively. Structural images were depicted by UCSF Chimera software ver. 1.13.1. (B and C) Transport activity of the BOR1 variants. (B) *S. cerevisiae bor1Δ* strains harboring AtBOR1 variants (WT, D311A, N355A, and Q360A) were spotted on SD-Ura +D-Galactose medium containing 0 or 15 mM boric acid and cultured for 6 days at 30°C. Diluted yeast suspensions were spotted onto the medium at OD_600_=0.4, 0.04, 0.004, or 0.0004. (C) ^11^B content in the yeasts after incubation with liquid medium containing 1 mM of boric acid for 50 min at 30°C. Different letters indicate significant differences between constructs, determined by ANOVA followed by Tukey–Kramer’s post hoc test (*P *<* *0.05). *n *=* *4 or 5 different colonies were used. Error bars represent mean ± SD.

To test this hypothesis, we sought additional amino acid residues that would directly play a role in B-transport. A previous report identified that the D347, N391, and Q396 amino acid residues, which surround the substrate-binding pocket of *S. cerevisiae* Bor1 (ScBor1), are important for its B-transport activity (Thurtle-Schmidt and Stroud, 2016). The D347 residue corresponds to E241 of the bacterial uracil transporter UraA and the E681 residue of human Band3/anion exchanger 1 (AE1; Supplemental Figure 7). The E681 residue of Band3/AE1 is considered to be the proton-binding site, which is essential for its transport activity (Reithmeier et al., 2016). The B-tolerance assay showed that the D347A substitution completely inhibited the B-transport activity of ScBor1, while N391A and Q396A partially inhibited it (Thurtle-Schmidt and Stroud, 2016). Because D347, N391, and Q396 of ScBor1 correspond to D311, N355, and Q360 of AtBOR1, respectively, their substitutions to alanine were expected to disturb the B-transport activity of BOR1 (Figure 4A;Supplemental Figure 7).

To investigate the relationship between the transport activity and ubiquitination of BOR1, we generated AtBOR1 variants carrying D311A, N355A, and Q360A substitutions and assessed their B-transport activity in yeast (Figure 4B and C). Immunoblotting and immunostaining against the Xpress-tag showed comparable expression levels and PM localization of the BOR1 variants in yeast cells (Supplemental Figures 6E–G). The B-tolerance assay showed that D311A completely inhibited the transport activity of BOR1, whereas N355A and Q360A did not or only slightly inhibited it (Figure 4B). Consistent with these results, the B-export assay showed that the D311A mutation completely inhibited the B-transport activity, whereas N355A and Q360A slightly inhibited it (Figure 4B and C, *P* <* *0.05 by one-way ANOVA followed by Tukey–Kramer’s posthoc test).

### Transport activity is necessary for high B-induced ubiquitination of BOR1

**Figure 5:**
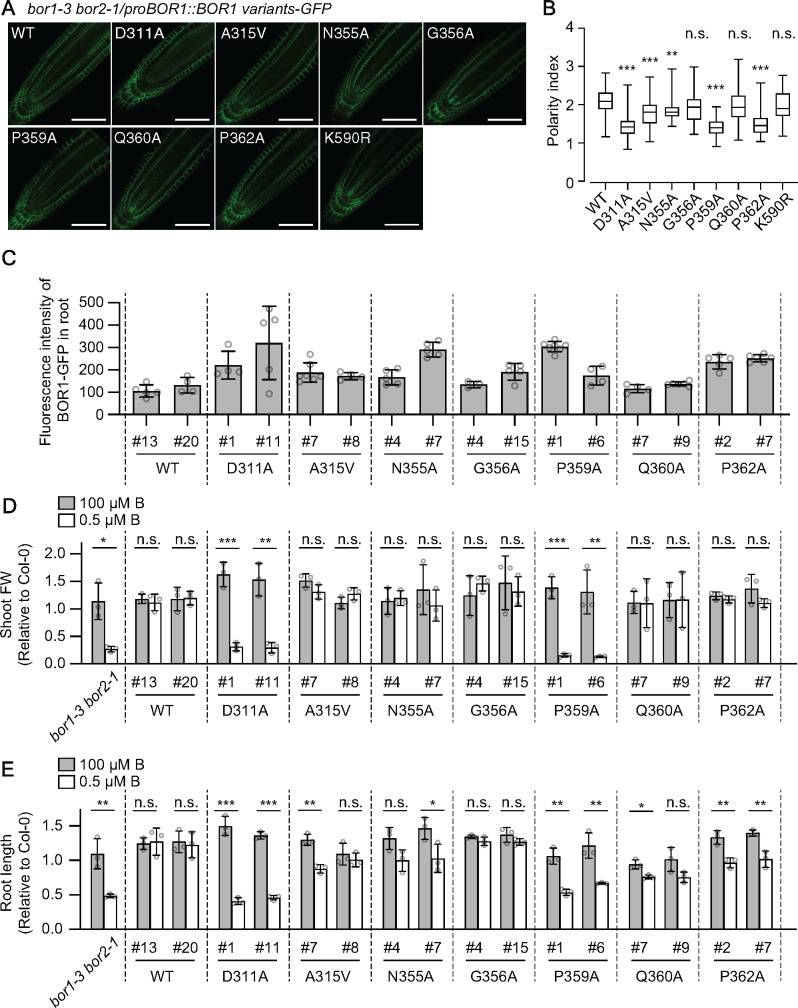
Boron-transport activity of BOR1 variants in plants. (A) Representative confocal images of BOR1-GFP variants in the primary root tip of Arabidopsis seedlings grown on 0.5 µM B MGRL medium for 5 days. Scale bars represent 50 µm. (B) Box plot representation of polarity indexes of BOR1-GFP variants. Letters above box plots indicate significant differences determined by the nonparametric Kruskal–Wallis test (*P <* *0.05, ***P <* *0.001, and ****P <* *0.0001, n.s. means no significant difference). *n *=* *49–87 cells from 3 to 5 different roots. Box plot center lines show the medians. Box limits indicate the 25th and 75th percentiles. Whiskers are extended to the highest and the lowest values. (C) Fluorescence intensity of BOR1-GFP. The total fluorescence of BOR1-GFP in the root tip was obtained by taking a Z-stack in 2 µm intervals over a total distance of 80 µm for each individual root by a 20× dry objective lens equipped with LSM800 (Zeiss). Error bars represent mean ± SD. *n *=* *4–8 plants. (D and E) Leaf fresh weight (D) and root length (E) values relative to WT Col-0 plants grown in the same plate. Error bars represent mean ± SD. Significant differences were determined by Student’s *t*-test (*P <* *0.05, **P <* *0.01, ***P <* *0.001). *n *=* *3 sets of plates.

To investigate the contribution of amino acid residues located in the substrate-binding pocket on B transport and degradation of BOR1 *in planta*, we introduced *proBOR1:BOR1-GFP* with D311A, N355A, and Q360A substitutions into *Arabidopsis thaliana*. We used a *bor1-3 bor2-1* double knockout mutant as background to avoid possible interaction between BOR1-GFP variants and endogenous BOR2 (Miwa et al., 2013). We also introduced *proBOR1:BOR1-GFP* with A315V, G356A, P359A, and P362A substitutions to validate the importance of the amino acid residues identified by the genetic screening using *pro35S:BOR1-GFP* constructs. All of the BOR1-GFP variants localized in the plasma membrane in a polar fashion toward the stele (Figure 5A). To assess the polar localization of BOR1-GFP variants, we calculated their polarity index in epidermal cells. Polarity indexes of G356A and Q360A variants were similar to that of WT BOR1-GFP, while D311A, N355A, P359A, and P362A variants showed reduced polarity of localization toward the stele (Figure 5B). To determine the effect of ubiquitination on the polar localization of BOR1, we also calculated the polarity index of the ubiquitination-deficient K590R variant. The value for the K590R variant was similar to that of WT BOR1-GFP (Figure 5B), indicating that a loss of ubiquitination does not affect polar localization of BOR1.

We then tested the functional complementation of the *bor1-3 bor2-1* phenotype by BOR1-GFP variants under a low-B condition. We selected lines with a similar or up to 2.4-fold higher accumulation of BOR1-GFP than the lines with WT BOR1-GFP in the root tip (Figure 5C). To quantify the plant growth, leaf-fresh weight, and root length relative to WT Col-0 grown in the same petri-dish was measured (Figure 5D;Supplemental Figure 8). Plant growth of *bor1-3 bor2-1* double mutants under a low-B condition was fully restored by introduction of WT BOR1-GFP (Figure 5D and E). As expected, the D311A and P359A variants whose B-transport activities were abolished or dramatically reduced in yeast cells, respectively (Figures 3E and 4C), did not restore the shoot and root growth of *bor1-3 bor2-1* (Figure 5D and E). The N355A and Q360A variants, which showed slight but significant reductions in the B-transport activity in yeast cells (Figure 4B and C), restored the shoot growth fully, but root growth only partially, in the low-B condition (Figure 5D and E). It is likely that the BOR1 variants partially defective in B transport complemented the shoot growth due to a relatively higher expression in transgenic plants. The G356A variants, which showed no or slightly reduced B-transport activity to WT in yeast cells (Figure 3D and E), fully restored the shoot and root growth (Figure 5D and E). The P362A variants, which showed no apparent reduction in the tolerance of yeast cells at a high B condition (Figure 3D and E), restored the shoot growth fully, but root growth partially (Figure 5D and E). The A315V variants, which showed similar B-transport activity to WT in yeast cells, fully restored the shoot and the root growth in a line (#8) but not fully in the other line (#7; Figure 5D and E). Taken together, our analyses in yeast and plants suggest that D311 and P359 are essential, N355 and Q360 are involved, and A315, G356, and P362 are less or not important for B-transport activity. The defective complementation of the root growth by the transport-competent A315V and P362A variants might be related to their reduced polar localization (Figure 5B).

**Figure 6:**
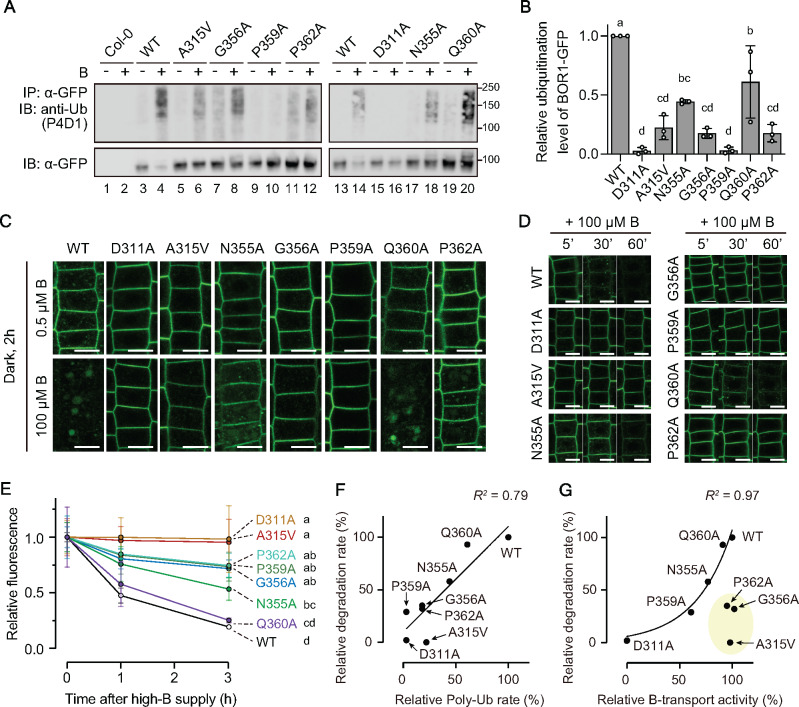
Vacuolar transport and ubiquitination of BOR1 variants. (A) Analysis of ubiquitination of BOR1-GFP variants by immunoblotting. Plants were grown with 100 µM B for 14 days followed by a shift to 0.5 µM B medium for 1 day. BOR1-GFP was immunoprecipitated from root-tissue-lysate extracted from the seedlings treated with 0.5 µM (−) or 500 µM (+) boric acid for 60 min. Ubiquitin and BOR1-GFP were detected by anti-ubiquitin monoclonal antibody (P4D1) and anti-GFP monoclonal antibody, respectively. As a negative control, WT Col-0 plants were used. (B) Relative ubiquitination level of the BOR1-GFP variants normalized by that of WT BOR1-GFP. Intensity of the ubiquitination band was divided by the corresponding GFP intensity. Bars represent mean ± SD. Dots indicate individual data points. *n = *3 independent replications. Different letters above plots indicate significant differences determined by one-way ANOVA followed by Tukey–Kramer’s post hoc test (*P* < 0.001). (C and D) Confocal images of root epidermal cells expressing the BOR1-GFP variants treated with 0.5 or 100 µM B. (C) Long-term incubation for 2 h under a dark condition to examine the increase of GFP in the vacuole. Scale bars indicate 10 µm. (D) Time course analysis of BOR1-GFP degradation. Scale bars indicate 10 µm. (E) Quantification of fluorescence intensity of BOR1-GFP variants treated with 100 µM boric acid for 0, 1, or 3 h. The total fluorescence of BOR1-GFP in the root tip was obtained by taking a *Z*-stack in 2 µm intervals over a total distance of 80 µm for each individual root by a 20× dry objective lens equipped with LSM800 (Zeiss). The intensities were compared to the treatment without the high-B treatment. Error bars represent mean ± SD. Different letters indicate significant differences between constructs, determined by two-way ANOVA followed by Tukey–Kramer’s post hoc test (*P *<* *0.01). *n *=* *4–7 plants. (F) Relationship between polyubiquitination level and degradation rate of BOR1-GFP variants. Plots showed linear correlation (*R^2^*=0.79). G, Relationship between B-transport activity and degradation rate of BOR1-GFP variants. The percentage relative to the value of BOR1 WT (Supplemental Table 1) was used for individual plots. Plots except A315V, G356A, and P362A showed exponential correlation (*R^2^*=0.97).

To address the relationship between B-transport activity and B-induced ubiquitination of BOR1, we examined whether the BOR1-GFP variants undergo polyubiquitination after high-B supply *in planta* using antibodies against general ubiquitin (P4D1) and K63-linked polyubiquitination (Apu3; Figure 6A;Supplemental Figures 9 and 10). The variants A315V, N355A, G356A, Q360A, and P362A showed reduced rates of polyubiquitination upon high-B supply compared to WT; 22 ± 10%, 44 ± 1.7%, 18 ± 4.2%, 61 ± 31%, and 18 ± 7.4% of WT, respectively (*P *<* *0.05 vs. WT by one-way ANOVA with Tukey–Kramer’s post hoc test), while D311A and P359A did not show such ladder-like signals; 2.8 ± 2.9% and 3.2 ± 2.7% of WT, respectively (Figure 6B).

We also tested whether the BOR1-GFP variants are degraded in response to high-B supply (Figure 6C–E; Supplemental Figure 11). The vacuolar sorting of BOR1-GFP was examined using the fact that GFP is relatively stable in the vacuole in the absence of light (Tamura et al., 2003; Takano et al., 2010). When the plants were treated with 100 µM B for 2 h in the dark, the WT and Q360A variant showed strong GFP fluorescence in the vacuole, while the N355A and P362A variants showed GFP fluorescence in punctate structures, presumably endosomes, and faint GFP fluorescence in the vacuole (Figure 6C). Additionally, we performed time course analysis under a normal light condition and quantified total GFP fluorescence in primary root tips (Figure 6D and E; Supplemental Figure 11). Within 60 min after 100 µM B supply, WT BOR1-GFP and N355A and Q360A variants showed a clear serial decrease of fluorescence, while D311A, A315V, G356A, P359A, and P362A showed relatively stable fluorescence in the plasma membrane (Figure 6D). A quantitation revealed that 19.3 ± 1.1% of WT BOR1-GFP fluorescence remained after 3 h (Figure 6E and Supplemental Figure 11). Q360A showed a fluorescence decrease rate comparable to WT; 25.3 ± 2.0% at 3 h (*P *=* *0.97 vs. WT by two-way ANOVA with Tukey–Kramer’s post hoc test). G356A, P359A, P362A, and N355A showed significantly slower rates of decrease than WT BOR1; 71.7 ± 7.8%, 76.3 ± 35%, 74.5 ± 11%, and 53.4 ± 10% at 3 h, respectively (*P *<* *0.0001 vs. WT by two-way ANOVA with Tukey–Kramer’s post hoc test). Strikingly, D311A and A315V variants did not show a decrease within 3 h (*P *>* *0.75 between time points by two-way ANOVA with Tukey–Kramer’s posthoc test).

The relationship between the B-dependent polyubiquitination, degradation, and the B-transport activity in yeast cells was analyzed based on their quantified values among the BOR1 variants (Figure 6F and G; Supplemental Table 1). As expected, the rates of B-dependent polyubiquitination and degradation showed a positive correlation, confirming that the polyubiquitination leads to vacuolar transport and degradation of BOR1 (Figure 6F). As an exception, the P359A variants showed degradation at a slightly smaller scale than WT, but no apparent ubiquitination could be observed. It is possible that ubiquitinated BOR1-GFP proteins are rapidly subjected to vacuolar transport and degradation, and the minor amount of the ubiquitinated P359A variant might be below the detection limit.

We then compared the B-transport activity in yeast cells and the rate of degradation (Figure 6G). The D311A variant was completely inactive in B-transport and not transported to the vacuole. The D311A, P359A, N355A, and Q360A variants and WT showed a positive correlation between the B-transport activity and the rate of degradation. These results support the hypothesis that substrate binding and the B-transport cycle of BOR1 are linked to B-induced ubiquitination. It should be noted that the A315V, G356A, and P362A variants were transport competent, but fully or partially defective in ubiquitination and degradation. These variants might have defects in specific conformations (see “Discussion” section).

**Figure 7:**
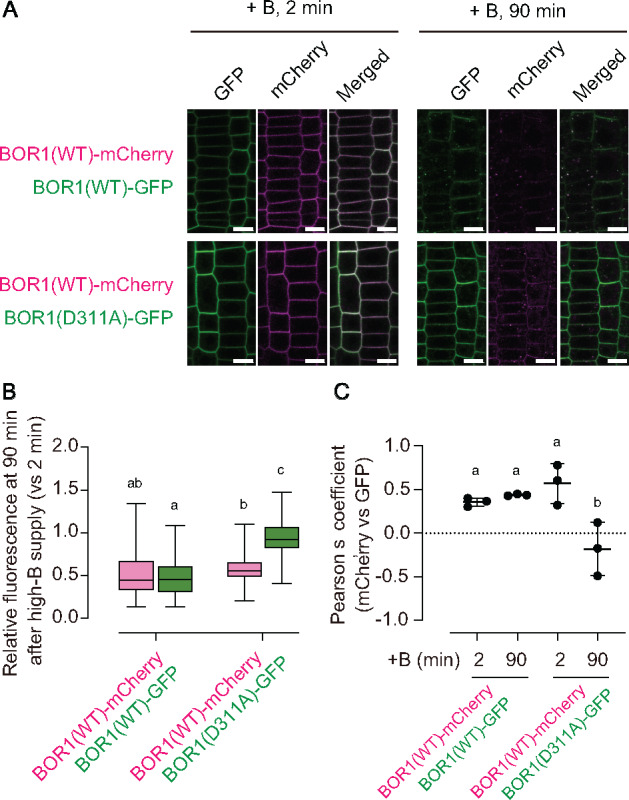
WT BOR1-GFP in the same cells does not promote degradation of BOR1(311A)-GFP. (A) F1 plants derived from the cross of *proBOR1:BOR1-GFP*/*bor1-1* or *proBOR1:BOR1(D311A)-GFP*/*bor1-3 bor2-1* and *proBOR1:BOR1-mCherry*/*bor1-1* were grown on solid medium containing 0.5 µM boron for 6 days and shifted to liquid MGRL medium containing 100 µM B for 2 or 90 min. Scale bars represent 10 µm. (B) Box plot representation of the relative fluorescence of BOR1(WT)-mCherry and BOR1(WT/D311A)-GFP in the plasma membrane compared with those at 2 min after high-B supply. *n *=* *90 cells from three different roots for each set. Letters above plots represent significant differences determined by one-way ANOVA with Tukey–Kramer’s post hoc test (*P *<* *0.01). (C) Pearson’s coefficient between GFP and mCherry fluorescence signals in the root epidermal cells treated with high-B for 2 or 90 min. *n *=* *3 images from different roots (panels in A are representative). Error bars indicate mean ± SD. Dots indicate individual data points. Different letters above plots indicate significant differences determined by one-way ANOVA with Tukey–Kramer’s post hoc test (*P *<* *0.05).

To rule out the possibility that the transport-defective variants do not show degradation because of the aberrant B concentrations inside or outside the cells, BOR1(D311A)-GFP and BOR1(WT)-mCherry were expressed in the same plants. In a control experiment, BOR1(WT)-GFP and BOR1(WT)-mCherry similarly showed endocytosis and degradation in the same epidermal cells within 90 min after 100 µM B supply (Figure 7A–C). By contrast, BOR1(D311A)-GFP was retained in the plasma membrane in cells where BOR1(WT)-mCherry was mostly transferred into endosomes (Figure 7A and B). BOR1(WT)-mCherry remained to a slightly higher extent in the cells with BOR1(D311A)-GFP than in those with BOR1(WT)-GFP (Figure 7B). This could be because of dimerization with each other. A quantification of the colocalization by Pearson’s correlation coefficient confirmed that BOR1(WT)-mCherry behaved similar to BOR1(WT)-GFP but differently compared to BOR1(D311A)-GFP (Figure 7C). These results suggest that B-transport activity rather than the B concentration inside or outside the cells controls B-induced endocytosis and degradation of BOR1.

The K590 residue is located closely to the last TM domain (Figure 2C) and its accessibility by an unknown ubiquitin ligase is expected to be influenced by conformational transition of BOR1 during B-transport. To test the importance of the position of the K590 residue, we generated BOR1-GFP constructs whose C-terminal regions including K590 were tandemly repeated (Supplemental Figure 12). If the ubiquitination of the K590 residue is independent of its position, the distal lysine residue in the extended C-terminal tail would be ubiquitinated in response to high-B supply and lead BOR1 to the vacuole. For convenience, the proximal (original) K590 residue is termed proximal K and the K590 residue in the repeated sequence is termed distal K. The C-terminal extended BOR1-GFP with proximal K (WT) was localized in the plasma membrane under the low-B condition and degraded in response to high-B supply regardless of the distal K presence (Supplemental Figure 12B and C; WT/WT, WT/K590R). The C-terminal extended BOR1-GFP with proximal K590R was localized in the plasma membrane under the low-B condition and it was not degraded in response to high-B supply regardless of the distal K presence (Supplemental Figure 12B and C; K590R/WT, K590R/K590R). Although the structure around the distal K is unclear, these data support the importance of the position of the ubiquitination site for B-induced degradation of BOR1. It is likely that conformational transition of BOR1 during B transport affects the local structure around the K590 residue that is close to the TM domain and provides a suitable environment for recognition by a ubiquitin ligase.

## Discussion

**Figure 8:**
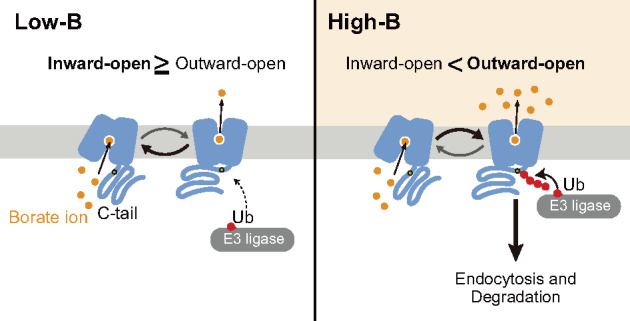
A model for B sensing. A transport-ubiquitination coupled model. Under low-B conditions, BOR1 is largely in the inward-open state, with the K590 residue in the C-tail unexposed. Under high-B conditions, BOR1 is more frequently in the outward-open state, with the C-tail exposed, giving access to the K590 residue for E3 ligases. Longer exposure to the E3 ligase results in polyubiquitination and subsequent endocytic degradation of BOR1.

Several plant transporters have been shown, or proposed, to have receptor functions and thus are called transceptors. For instance, the Arabidopsis dual-affinity nitrate transporter CHL1/NRT1.1 functions in nitrate sensing, which controls the induction of nitrate-responsive genes including a high-affinity nitrate transporter *NRT2.1* (Ho et al., 2009); the nitrate sensing and transport functions of CHL1/NRT1.1 can be decoupled by a mutation. In addition, the Arabidopsis iron transporter IRT1, which transports various metals and is degraded upon noniron metal stress, contains a histidine-rich stretch in the cytosolic loop region that directly binds noniron metals, is phosphorylated by the CIPK23 kinase, and subsequently is ubiquitinated by IDF1, a RING-type E3 ubiquitin-ligase (Dubeaux et al., 2018). Substitution of four histidines to alanines (4HA) in the histidine-rich stretch does not affect IRT1 transport activity but diminishes its phosphorylation and ubiquitination. Importantly, the expression of IRT1(4HA) made plants hypersensitive to noniron metal excess. These results indicate that the metal-binding site of IRT1 regulates its own degradation to protect plants from metal stress (Dubeaux et al., 2018). In contrast to these examples, our study showed that the residues required for borate transport activity of BOR1 were also necessary for the B-sensing mechanism regulating its own degradation (Figure 8). We, therefore, propose that BOR1 is a transceptor for the maintenance of B homeostasis with a different sensing mechanism from known plant transceptors.

**Figure:**
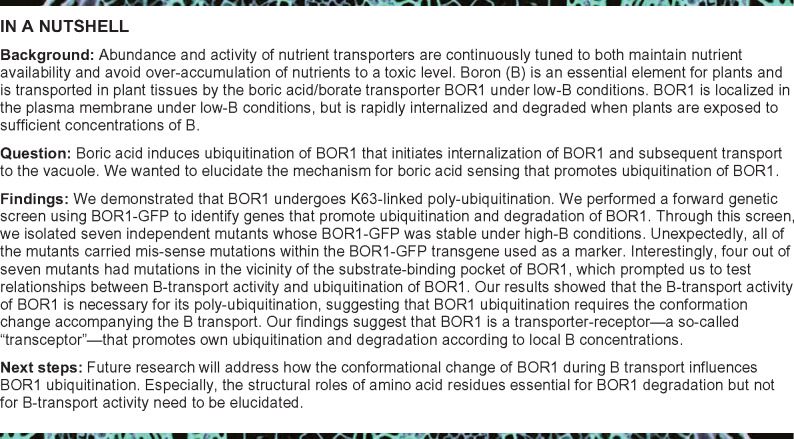


In fungi, many nutrient transporters are downregulated via ubiquitination, subsequent endocytosis, and degradation in the vacuole under substrate supply at high concentrations (Babst, 2020). In budding yeast *S. cerevisiae*, arrestin-related adaptors, Arts, recruit a ubiquitin ligase Rsp5 to specific transporters in the plasma membrane for ubiquitination (Lin et al., 2008). Amino acid permeases such as Gap1, Mup1, Fur4, and Can1 harbor degrons in their cytosolic tail regions (Keener and Babst, 2013; Ghaddar et al., 2014; Guiney et al., 2016). It was proposed that the specific degron, where Arts and Rsp5 are recruited to, is masked at a ground state (state under a low substrate concentration), while it gets exposed at an activated state (state under high substrate concentration) due to a conformational transition accompanied with substrate transport (Guiney et al., 2016). BOR1 is distantly related to the amino acid permeases in the same amino acid-polyamine organocation (APC) superfamily, and they share an overall architecture (Vastermark et al., 2014). Therefore, it is reasonable that similar mechanisms of transport-coupled ubiquitination operate in the downregulation of yeast amino acid permeases and plant BOR1.

In this study, we first addressed the characteristics of BOR1 ubiquitination and observed that BOR1 undergoes K63-linked polyubiquitination in a B-dependent manner. Lysine 63-linked polyubiquitination is the second most abundant type of polyubiquitination and functions in a variety of cellular processes in eukaryotes (Liu et al., 2018). In plant cells, abundance of membrane proteins in the plasma membrane such as IRT1, the auxin transporter PIN2, and the brassinosteroid receptor BRI1 are controlled by accelerated endocytosis and/or vacuolar transport dependent on K63-linked polyubiquitination (Leitner et al., 2012; Martins et al., 2015; Dubeaux et al., 2018). In BOR1 protein, the K590 residue located in the cytosolic C-tail region is essential for B-dependent ubiquitination and vacuolar transport, while there was no evidence of direct ubiquitination of the residue (Kasai et al., 2011). Although BOR1 was described to undergo mono- or diubiquitination in the previous study (Kasai et al., 2011), we detected ladder-like signals recognized by a specific antibody against K63-linked ubiquitination (Figure 1D). Furthermore, we demonstrated that the K590 residue is directly modified with ubiquitin by MS analysis (Figure 1C). These results suggest that BOR1 undergoes K63-linked polyubiquitination at the K590 residue. Recently, we demonstrated that the rapid endocytosis of BOR1 for high B-induced vacuolar transport is independent of the AP-2 clathrin adaptor complex (Yoshinari et al., 2019). These findings imply that an unknown ubiquitin-binding endocytic adaptor binds to K63-linked polyubiquitin attached to BOR1. A recent study proposed that TOM1-LIKE6 (TOL6), a plasma membrane-associated ubiquitin receptor, and its paralogs act as K63-linked ubiquitin adaptors facilitating vacuolar transport of plasma membrane proteins (Moulinier-Anzola et al., 2020). Indeed, vacuolar transport of BOR1 is compromised in a higher order mutant of *TOL*s (*tol23569*; Yoshinari et al., 2018). TOL proteins associated with the plasma membrane might recognize BOR1 with K63-linked polyubiquitin for vacuolar transport.

Recently, crystal structural analyses revealed the three-dimensional structure of BOR1 in an occluded conformation at a 4.1 Å resolution and ScBor1p in an inward-open conformation at a 5.9 Å resolution (Coudray et al., 2016; Thurtle-Schmidt and Stroud, 2016). The structure of BOR1 can be distinguished into two domains, the “gate domain” and “core domain”. The gate domain is regarded as a rigid scaffold and is required for oligomerization, while the core domain moves to achieve the rocking bundle (Coudray et al., 2016) or the elevator transport mechanism (Thurtle-Schmidt and Stroud, 2016). In the core domain, the borate anion is thought to be stabilized in the space between the positive dipoles, TMD 3 and 10 (Figure 4A;Supplemental Figure 7). Our genetic screen using BOR1-GFP identified G201, V250, and S251 residues located in the gate domain as important residues for ER exit (Supplemental Figure 4). These amino acid residues are probably essential for proper protein folding or structural stability of BOR1. In the same genetic screen and targeted mutagenesis, we determined that mutations at A315, D311, N355, G356, P359, Q360, and P362 disturbed ubiquitination and vacuolar transport of BOR1 to different degrees (Figures 2–6). These amino acid residues are located around the N-terminus of TMD 10 which composes the putative borate-binding pocket in the core domain (Figure 4A;Supplemental Figure 7). Intriguingly, a substitution of the putative proton-binding amino acid residue D311 in the TMD 8 completely abolished transport activity, ubiquitination, and vacuolar transport of BOR1 (Figures 4–6). The transport-deficient D311A variant was not transported to the vacuole when B transport was secured by the WT BOR1 expressed in the same cells (Figure 7). Furthermore, a positive correlation was observed between B-transport activity, ubiquitination, and degradation among the transport-defective D311A, P359A, N355A, and Q360A variants and WT (Figure 6F and G; Supplemental Table 1). By analogy to yeast amino acid permeases, these results suggest that the degradation of BOR1 is via transport-coupled ubiquitination (Figure 8). When the extracellular concentration of borate/boric acid is relatively low, the transport cycle would proceed as follows: (1) The borate anion accesses the substrate-binding site during the inward-open state, (2) the conformation of BOR1 changes to the occluded state and then to the outward-open state, (3) the borate anion is released from the substrate-binding pocket and the conformation immediately returns to the inward-open state. However, when the extracellular concentration of borate/boric acid is relatively high, the borate anion would remain longer in the substrate-binding pocket to keep the outward-open state. We speculate that an unknown E3 ubiquitin ligase can access and conjugate the polyubiquitin chain to the K590 residue, which is exposed to the cytosol only in the outward-open state (Figure 8).

In our mutant analysis, the A315V, G356A, and P362A mutations did not strongly affect the B-transport activity in yeast, but inhibited ubiquitination and vacuolar transport of BOR1 (Figures 3 and 6). Presumably, these mutations affected specific structures of BOR1 that are involved in the ubiquitination but play only a minor role in B-transport. According to an in silico prediction, the A315V substitution leads to the formation of an aberrant hydrogen bond between the valine residue and S361 (Supplemental Figure 13). This abnormal hydrogen bond may affect the conformation of BOR1 in the outward-open state without inhibiting the B-transport activity. We speculate that valine at this position might add a specific physiological function to certain BOR1 homologs, because the sequence is observed in AtBOR5 and plant orthologs (Figure 3C;Supplemental Figure 5). Interestingly, D311A, A315V, N355A P359A, and P362A mutations affected polar localization of BOR1 toward the stele in addition to ubiquitination (Figures 5B). These mutations might affect the structure of the later C-tail region (637–704 aa), which is recognized by the AP-2 clathrin adaptor complex and is required for polar localization of BOR1 (Kasai et al., 2011; Yoshinari et al., 2019), in addition to the region proximal to the last TMD. Further analysis is required to understand the structural impact of the mutations on the cytosolic C-tail movement during the transport cycle.

In conclusion, our study revealed that the ubiquitination and degradation of the BOR1 transporter are coupled to the borate transport cycle. We propose that BOR1 is a B transceptor and is capable of rapid self-regulation. This transport-coupled ubiquitination model for nutrient transceptors appears to be conserved in organisms including plants and fungi to cope with a sudden increase of nutrient availability.

## Materials and methods

### Plant materials

To construct *35S promoter:BOR1-GFP*, the *BOR1-GFP* sequence in a pAT30 entry clone (Takano et al., 2010) was subcloned into pMDC32 (Curtis and Grossniklaus, 2003), a gateway destination vector containing a dual *35S* promoter, by LR recombination (Invitrogen). The resulting construct was introduced into *Arabidopsis thaliana* Col-0 using the Agrobacterium-mediated floral dip method (Clough and Bent, 1998). Mutant constructs carrying A315V, G356A, P359A, P362A, D311A, N355A, Q360A, or K590R were generated by site-directed mutagenesis into pAT30 (*BOR1-GFP* in pENTR D-TOPO) using primers listed in Supplemental Table 2 and subsequent LR recombination with pAT100, a gateway destination vector containing *proBOR1* (Takano et al., 2010). The *proBOR1:BOR1-GFP* construct (Takano *et al.*, 2010) and its mutant constructs were introduced into the *Arabidopsis thaliana bor1-3 bor2-1* double knockout mutant (Kasai et al., 2011). For construction of the C-terminally extended BOR1-GFP, DNA fragments corresponding to the BOR1 C-terminal region were amplified from entry vectors containing WT and K590R-mutated *BOR1-GFP* (*BOR1[WT or K590R]-GFP* in pENTR D-TOPO), and the entry vectors were linearized by inverse PCR using primers listed in Supplemental Table 2. The DNA fragments for the C-terminal region were integrated into the linearized *BOR1-GFP* entry vectors by the In-Fusion technique (Clontech). For observation of BOR1(WT/D311A)-GFP and BOR1(WT)-mCherry in the same cells, the F1 generation plants of a cross between BOR1(WT/D311A)-GFP*/bor1-3 bor2-1* and BOR1-mcherry/*bor1-1* (Miwa *et al.*, 2013) were used. Plant materials used in this study were also listed in Supplemental Data Set 2.

### Plant growth conditions

Seeds were surface-sterilized with 70% (v/v) ethanol, washed with sterile water four times, and sown on modified MGRL medium (Takano et al., 2005) solidified with gellan gum (Wako Pure Chemicals, Osaka, Japan) in plastic plates. The plates were incubated at 4°C for 3 days and placed vertically in the plant growth chamber at 22°C with 16 h at a light intensity of ∼120 µmol m^−2^ s^−1^ (emitted by white fluorescent lamps) and 8 h dark. The concentration of boric acid in the media is described in each figure legend.

### Confocal microscopy and quantification of fluorescence of plants

Confocal images of BOR1-GFP expressed in roots were with confocal laser scanning microscopes, Leica TCS-SP8, Zeiss LSM510, or Zeiss LSM800, equipped with a 40× water-immersion lens or a 63× oil-immersion lens. GFP was excited with a 488 nm wavelength by a diode or argon laser and detected with a 500–530 nm (SP8) or 505–550 nm (LSM800) window of wavelength. For visualization of the ER, seedlings were incubated with 4 µM ER Tracker Red (Invitrogen, CA) for 5 min. ER Tracker Red was excited with a 561 nm wavelength of a diode laser and detected with a 600–650 nm window of wavelength (SP8).

For the time course analysis of BOR1-GFP WT and K590R (Figure 1), seedlings were placed in a cover glass bottom chamber (D11131H, Matsunami, Osaka, Japan), and the roots were covered with MGRL medium containing 1% (w/v) gellan gum and 100 µM boric acid. Images were taken at the indicated time points using a TCS SP-8 system (Leica). The fluorescence of BOR1-GFP in the plasma membrane was quantified by Fiji/ImageJ software as described previously (Yoshinari and Takano, 2020).

For the time course analysis of BOR1-GFP variants harboring amino acid substitutions in the vicinity of the substrate-binding pocket (Figure 6) and C-terminally extended BOR1-GFP variants (Supplemental Figure 12), seedlings were grown for 5 days on modified MGRL medium with 0.5 µM B and then floated on liquid MGRL medium containing 100 µM boric acid for 0, 60, and 180 min. The total fluorescence of BOR1-GFP in the root tip was obtained by taking a Z-stack in 2 µm intervals over a total distance of 80 or 100 µm for each individual root with an LSM800 (Zeiss) using a 20× dry objective lens or a TCS-SP8 (Leica) using a 40× water-immersion objective lens. The fluorescence of BOR1-GFP in the sum image of the root tip region (see Supplemental Figure 11) was quantified by Fiji/ImageJ software. Comparison of expression levels of BOR1-GFP variants under a low-B condition (Figure 5A) was performed in a similar way.

The polarity index was calculated as described previously (Yoshinari and Takano, 2020). Briefly, confocal images of BOR1-GFP in the epidermal cells of the meristematic to transition zones in primary root tips were used for the calculation. Plasma membrane regions at the apical and basal domains were selected with 3-pixel-wide straight lines by Fiji/ImageJ software. The integrated density (total fluorescence) in the inner half of the plasma membrane was divided by that in the outer half of each cell.

For the analysis of high B-induced degradation of BOR1-mCherry and BOR1 (WT/D311A)-GFP (Figure 7), seedlings were grown for 5 days on modified MGRL medium with 0.5 µM B and then floated on liquid MGRL medium containing 100 µM boric acid for 2 or 90 min. The fluorescence of mCherry and GFP in the plasma membrane was observed with a TCS-SP8 (Leica) and quantified as described previously (Yoshinari and Takano, 2020). Pearson’s correlation coefficient was calculated using Fiji/ImageJ with the PSC Colocalization plugin (French et al., 2008).

### Mutant screening

For the mutant screening, approximately 12,500 T4 seeds of *pro35S:BOR1-GFP* were mutagenized with 0.3% ethylmethanesulfonate (EMS) overnight and two pools of M2 seeds were obtained. M2 seedlings were grown on solid medium containing 500 µM B for 7 days. GFP images were visualized by the macro zoom microscope MVX-10 (Olympus) with a CCD camera CoolSNAP EZ (Photometrics).

### LC-MS/MS analysis of BOR1-GFP protein

Total proteins were extracted from 22-day-old transgenic plants harboring *pro35S:BOR1-GFP* (-5′UTR) grown with 0.5 µM B. The homogenization was carried out in buffer (250 mM Tris-HCl [pH 8.5], 290 mM sucrose, 1 mM Pefabloc^®^ SC [Roche], 1.25% [v/v] PSC-protector solution [Roche], cOmplete ULTRA Mini EDTA-free [Roche], 5 mM DTT) with a multibeads shocker (Yasui Kikai, Osaka, Japan). Cell debris was removed by centrifugation at 10,000g at 4°C for 15 min three times. The microsome fraction was isolated from the supernatant by ultracentrifugation (100,000 g, 10 min, 4°C) and resuspended in lysis buffer (150 mM NaCl, 1% [v/v] Triton X-100, 50 mM Tris-HCl [pH 8.0], 1% [v/v] 3-[{3-cholamidopropyl}dimethylammonio)-1-propanesulfonate [CHAPS]) using a Potter-Elehjem tissue grinder. BOR1-GFP was isolated by µMACS™ anti-GFP microbeads (Miltenyi Biotec) according to the manufacturer’s instructions. BOR1-GFP protein was collected together with the microbeads in the lysis buffer by dissociating the column from the µMACS™ Separator magnet. The protein–microbead suspension was incubated with the lysis buffer containing 230 µM B on ice for 30 min right before SDS-PAGE. Immunoprecipitated proteins were separated by NuPAGE 4%–12% Bis-Tris Gel (Invitrogen) and stained with Flamingo fluorescent gel stain solution (Bio-Rad). The band of the BOR1-GFP monomer was cut and placed into acetonitrile. The gel was dried in a vacuum concentrator and treated with a reducing solution (10 mM DTT, 50 mM ammonium bicarbonate) at 56°C for 15 min. After supernatant removal, the gel was treated with alkylation solution (55 mM 2-iodoacetamide, 50 mM ammonium bicarbonate) for 30 min. The supernatant was removed again, and the gel was washed with 50 mM ammonium bicarbonate three times and dried up in a vacuum concentrator. After drying, the gel was treated with an enzyme solution (10 µg/mL chymotrypsin sequencing grade [Roche], 100 mM Tris-HCl [pH 7.9], 10 mM CaCl_2_) overnight at 25°C. The supernatant was collected and concentrated by vacuum centrifugation. To remove debris, the protein solution was filtered with an Ultra free-MC 0.45 µm Filter unit (Merck Millipore). Modification of BOR1-GFP was detected by Thermo Scientific Q Exactive Plus Orbitrap LC-MS/MS (Thermo Scientific). The MS/MS spectra were analyzed by Thermo Scientific Proteome Discoverer with MASCOT (Matrix Science, MA) and Sequest HT (Thermo Scientific) algorithms.

### Immunoblot analysis of ubiquitination

For immunoprecipitation of BOR1-GFP, transgenic plants harboring *proBOR1:BOR1-GFP* were used. Shoot tissues were removed, and root tissues were harvested. Root tissues (0.2–0.6 g) were lysed in 0.5–2.0 mL of extraction buffer (50 mM Tris-HCl pH7.5, 5 mM DTT, 100 mM NaCl, 10% [v/v] glycerol, 2% [v/v] IGEPAL, 20 mM N-ethylmaleimide, complete mini EDTA-free [Roche], 0.25 mg/mL Pefabloc^®^ SC) by using a mortar and a pestle or a multibeads shocker. Cell debris was removed by centrifugation at 10,000g at 4°C for 15 min three times. Anti-GFP IP was carried out following the manufacturer’s instructions (Miltenyi Biotec) with slight changes. Immunoprecipitated proteins were eluted with 70 µL of preheated elution buffer (50 mMTris-HCl [pH 6.8], 1% [w/v] SDS, 0.005% [w/v] Bromophenol Blue, 10% [v/v] glycerol, and 100 mM DTT, 95°C). Immunoprecipitated proteins were separated by Bolt™ 4%–12% Bis-Tris SDS-PAGE gel (Invitrogen) or NuPAGE^®^ 4%–12% Bis-Tris SDS-PAGE gel (Invitrogen) with MOPS buffer and then transferred to an Immobilon PVDF membrane. Anti-ubiquitin mouse monoclonal antibody, P4D1 (Cat#: sc-8017, Lot#: F2320 or sc-8017 HRP, Lot#: A0919 [Santa Cruz Biotechnology], 1:2,000 dilution), anti-GFP monoclonal antibody (Cat#: 4363-24, Lot#: M9R8752 [Nacalai Tesque, Kyoto], 1:2,000 dilution), anti-ubiquitin, K63 specific, Apu3 (Cat#: 05-1308, Lot#: 3137755 [Merck], 1:2,000 dilution), and anti-ubiquitin K48 specific, Apu2 (Cat#: 05-1307 [Merck], 1:1,000 dilution) were diluted in Can Get Signal Solution 1 (Toyobo, Osaka, Japan). Anti-mouse IgG HRP-conjugated antibody (Cat#:115-035-003, Lot#: 138339 [Jackson ImmunoResearch, PA], 1:200,000 dilution or Cat#: NA931, Lot#: 16810265 [GE Healthcare], 1:20,000 dilution) and anti-rabbit IgG HRP-conjugated antibody (Cat#: 111-035-144, Lot#: M9M6770 [Jackson ImmunoResearch]) were diluted in Can Get Signal Solution 2 (Toyobo). For detection of luminescence, Luminata Forte Western HRP substrate (Millipore) was used.

For quantification of polyubiquitination rates, ladders in 100–250 kDa detected by an anti-ubiquitin antibody (P4D1) and single bands (∼98 kDa) detected by an anti-GFP antibody were defined as polyubiquitination and nonubiquitinated BOR1-GFP signals, respectively. Mean intensities of the area (100–250 kDa for polyubiquitination; ∼98 kDa for nonubiquitinated BOR1-GFP) were quantified by Fiji/ImageJ software (Schindelin et al., 2012). Background signals (from a nonloaded region) were subtracted before calculation. The mean intensity of the polyubiquitination was divided by the mean intensity of the corresponding nonubiquitinated BOR1-GFP.

### Boron transport assay in yeast

The *S. cerevisiae* *bor1⊿* strain Y01169 (MAT*a his3 leu2 met15 ura3 YNL275W::kanMX4*; Takano et al., 2007), which lacks endogenous B export activity was used. To construct the vector to express BOR1 in yeast, BOR1 CDS was amplified using a BOR1 cDNA as a template with specific primers (Supplemental Table 2). The cDNA was subcloned into the pYES2 NT/A vector (Invitrogen) digested by KpnI and XhoI using the In-Fusion technique (Clontech). Constructs carrying A315V, G356A, P359A, P362A, D311A, N355A, or Q360A mutations were generated by site-directed mutagenesis of pTS32 (*BOR1* in pYES2 NT/A) using primers listed in Supplemental Table 2. To determine the B export activity, an empty vector control (pYES2/NT-A) or constructs with respective AtBOR1 variants (WT, D311A, A315V, N355A, G356A, P359A, and P362A) were introduced into the strain and selected on SD-Uracil + Raffinose media (2% [w/v] raffinose, 6.7 g/L yeast nitrogen base with ammonium sulfate, 20 mg/L of L-Histidine, 100 mg/L of L-Leucine, and 20 mg/L of L-Methionine, 2% [w/v] agar). Five colonies per construct were incubated for 23 h at 30°C in SD-Uracil + Raffinose liquid media without agar. 1.2 mL of the preculture were inoculated into 30 mL SD-Uracil + Galactose media (2% [w/v] D-galactose, 6.7 g/L yeast nitrogen base with ammonium sulfate, 20 mg/L of L-Histidine, 100 mg/L of L-Leucine, and 20 mg/L of L-Methionine) and incubated at 28°C with shaking. At an OD_600_ between 0.4 and 0.6, samples were diluted to an equal OD_600_ of 0.4 in 30 mL volume. Cells were collected by centrifugation and resuspended in 30 mL of fresh SD-Uracil + Galactose containing 1 mM boric acid. Then the cells were incubated at 30°C for 50 min under mild shaking. After incubation, the cells were immediately cooled in an ice bath and collected by centrifugation at 4°C. The cells were washed by ice-cold water three times and subsequently collected by centrifugation at 4°C. Cell pellets were dried up at 70°C in an oven for 3 days. Cell pellets with large differences in dry weight were excluded from the measurement. The cell pellets were digested with 60% HNO_3_ and dissolved in 0.08 M HNO_3_ containing 5 ppb beryllium as an internal standard. The ^11^B concentration in the sample was measured by inductively coupled plasma mass spectrometry (ICP-MS, Agilent 7800).

For the spotting assay, yeast cells were grown in 3 mL of SD-Ura + Raffinose liquid medium until OD_600_ = ∼0.7 and diluted to OD_600_ = 0.4. Then 10 µL volumes of a 10 times dilution series were spotted on SD-Ura + Galactose solid medium containing 2% (w/v) agar and 0, 10, or 20 mM boric acid and incubated at 30°C for 6 days.

### Extraction and immunoblot analysis of yeast proteins

Yeast cells were initially grown in 3 mL of SD-Ura + Raffinose liquid media at 30°C overnight with shaking. The 3 mL of fresh SD-Ura + Galactose liquid media were inoculated with 100 µL of the culture media and incubated at 28°C overnight with shaking. For the high-B shift assay, the same amount of yeast cells expressing WT-BOR1 was incubated in SD-Ura +Galactose liquid media containing 0, 10, or 20 mM boric acid for 1 h right before the following procedure.

The 1 mL of yeast cells (OD_600_ = 1.0) was transferred to 1.5 mL tubes and collected by centrifugation at 15,000 g for 1 min. After removal of the supernatant, cell pellets were suspended in 100 µL of ice-cold protease inhibitor mix (2 mM phenylmethylsulfonyl fluoride [PMSF], 2× complete protease inhibitor cocktail [Roche], 8 mM EDTA). Then, 50 µL of a 2 M NaOH solution was added to the cell suspension. After incubation for 10 min on ice, 50 µL of 50% trichloroacetic acid (TCA) solution was added, and cells were incubated for 10 min on ice. Samples were centrifuged at 15,000 g for 5 min at 4°C. After removal of supernatant, cell pellets were resuspended in 50 µL of sample buffer 1 (100 mM Tris-HCl [pH 6.8], 4 mM EDTA, 4% SDS, 20% glycerol, 0.02% bromophenol blue). Finally, 50 µL of sample buffer 2 (1 M Tris base, 2% β-mercaptoethanol) was added and heated at 98°C for 3 min.

Equivalent volumes of the samples were separated by SDS-PAGE using a 10% acrylamide gel with Tris-glycine running buffer. Proteins were transferred to an Immobilon PVDF membrane (Merck Millipore) with Tris-glycine/methanol transfer buffer by a semi-dry blotting method. Anti-Xpress tag mouse monoclonal antibody (Invitrogen, 1:5,000 dilution) and anti-mouse IgG HRP-conjugated antibody (Jackson Immuno Research, 1:100,000 dilution) were used as primary and secondary antibodies, respectively. For the dilution of antibodies, Can Get Signal solutions (Toyobo, Osaka, Japan) were used. Total proteins were stained with Coomassie Brilliant Blue (CBB).

### Yeast immunofluorescence analysis

Yeasts in the logarithmic growth phase in SD + Galactose liquid medium were fixed with 3.75% (w/v) paraformaldehyde for 30 min at 30°C with shaking. Cells were washed with 0.1 M potassium phosphate buffer (pH 7.5) and then with 1.2 M sorbitol/0.1 M potassium phosphate buffer (pH 7.5). To lyse the cell wall, cells were incubated with 2.5 mM DTT for 10 min at 30°C and then with 150 µg/mL zymolyase (Nacalai Tesque) and 0.5% 2-mercaptoethanol for 10 min at 30°C. Cells were collected by centrifugation and washed with 1.2 M sorbitol/0.1 M potassium phosphate buffer (pH 7.5). The 50 µL of cell suspension was put onto a MAS-coated slide glass (Matsunami Glass, Osaka, Japan) and treated with ice-cold methanol for 6 min and then by ice-cold acetone for 30 min. The sample on the sliding glass was dried completely and stored at room temperature. Before immunostaining, cells were rehydrated with wash buffer (5 mg/mL bovine serum albumin, 1X PBS). As a primary antibody, mouse anti-Xpress tag antibody (Invitrogen, 1:1,000) was added and incubated overnight at 4°C. The primary antibody was removed and the sample was washed with 100 µL of wash buffer four times. As a secondary antibody, anti-mouse IgG antibody conjugated with CF568 (Biotium, 1:1,000) was added and incubated for 2 h at 37°C. Cells were washed with 100 µL of wash buffer four times and with 1X Phosphate-buffered saline (PBS) three times. Confocal imaging of yeast cells was performed with a Leica TCS-SP8 equipped with a 63× glycerol immersion lens. For excitation and detection of CF568 signals, 552 nm and 560–650 nm wavelengths were used, respectively.

### Multiple alignment and phylogenetic analysis

A neighbor-joining phylogenetic tree of the BOR family based on protein sequences was constructed using a multiple sequence alignment of the MUSCLE algorithm in the MEGA X software (version 10.1.8; Molecular Evolutionary Genetics Analysis). Probabilities (%) of 1,000 bootstrap trials for each node were labeled in the tree. *Homo sapiens* AE 1 (HsAE1) was used as the outgroup on the basis of the structural similarity to BORs.

## Statistics

For statistical analysis, we used Prism 8 software (version 8.4.2; GraphPad Software, CA). Detailed ANOVA and *t*-test results are described in Supplemental Data Set 3.

## Accession numbers

Gene models used in this article can be found in the Arabidopsis Genome Initiative database under the following accession numbers: BOR1 (AT2G47160), BOR2 (AT3G62270), and NIP5;1 (AT4G10380).

## Supplemental data

**Supplemental Figure 1** LC-MS/MS analysis.

**Supplemental Figure 2** Additional data of BOR1-GFP ubiquitination.

**Supplemental Figure 3** K48-linked ubiquitination of BOR1-GFP was not detected.

**Supplemental Figure 4** Ectopic localization of G201R, V250F, and S251F mutants of BOR1-GFP.

**Supplemental Figure 5** Phylogenetic analysis of BOR family proteins.

**Supplemental Figure 6** Expression of Xpress-His6-BOR1 variants in yeast.

**Supplemental Figure 7** Comparison of substrate-binding pockets of AtBOR1, UraA, and Band3/AE1.

**Supplemental Figure 8** Growth phenotype of transgenic plants expressing BOR1-GFP variants.

**Supplemental Figure 9** Replication data of immunoblotting used for quantification of ubiquitination.

**Supplemental Figure 10** K63-linked polyubiquitination is affected in BOR1-GFP variants.

**Supplemental Figure 11** Total fluorescence of BOR1-GFP variants in the primary root tips.

**Supplemental Figure 12** C-terminal tail including K590 is not sufficient for B-induced degradation of BOR1.

**Supplemental Figure 13** In silico prediction of the A315V substitution impact on BOR1 structure.

**Supplemental Table 1** Relative B-transport activity, polyubiquitination, and B-induced degradation rate of the BOR1-GFP variants.

**Supplemental Table 2** Primer list.

**Supplemental File 1** Text file of the alignment used for the phylogenetic analysis shown in Supplemental Figure 5.

**Supplemental Data Set 1** Coordinates for phylogenetic analysis shown in Supplemental Figure 5.

**Supplemental Data Set 2** Plant materials.

**Supplemental Data Set 3** ANOVA and *t*-test results.

## Supplementary Material

koaa020_Supplementary_Data

## Notes

The author responsible for distribution of materials integral to the findings presented in this article in accordance with the policy described in the Instructions for Authors (www.plantcell.org) is: Junpei Takano (jtakano@plant.osakafu-u.ac.jp)

A.Y. and J.T. designed research; A.Y., T.H., M.P.B, K.O., Y.O., C.H., and T.E.T. performed experiments; A.Y., T.H., and K.O. analyzed data; C.H., T.E.T., Y.F., T.F., and J.T. supervised experiments; A.Y., M.P.B., and J.T. wrote the article.

## References

[koaa020-B1] Aibara I , Hirai T, Kasai K, Takano J, Onouchi H, Naito S, Fujiwara T, Miwa K (2018) Boron-dependent translational suppression of the borate exporter *BOR1* contributes to the avoidance of boron toxicity. Plant Physiol 177: 759–774 29728453 10.1104/pp.18.00119 PMC6001339

[koaa020-B2] Babst M (2020) Regulation of nutrient transporters by metabolic and environmental stresses. Curr Opin Cell Biol 65: 35–41 32200208 10.1016/j.ceb.2020.02.009 PMC7501145

[koaa020-B3] Barberon M , Zelazny E, Robert S, Conéjéro G, Curie C (2011) Monoubiquitin-dependent endocytosis of the transporter controls iron uptake in plants. Proc Natl Acad Sci USA 108: E450–E458 21628566 10.1073/pnas.1100659108 PMC3156158

[koaa020-B4] Bayle V , Arrighi J-F, Creff A, Nespoulous C, Vialaret J, Rossignol M, Gonzalez E, Paz-Ares J, Nussaume L (2011) *Arabidopsis thaliana* high-affinity phosphate transporters exhibit multiple levels of posttranslational regulation. Plant Cell 3: 1523–35 10.1105/tpc.110.081067 PMC3101552 21521698

[koaa020-B5] Clough SJ , Bent AF (1998) Floral dip: a simplified method forAgrobacterium-mediated transformation of *Arabidopsis thaliana*. Plant J 16: 735–743 10069079 10.1046/j.1365-313x.1998.00343.x

[koaa020-B6] Coudray N , Seyler L S, Lasala R, Zhang Z, Clark KM, Dumont ME, Rohou A, Beckstein O, Stokes DL (2016) Structure of the SLC4 transporter Bor1p in an inward-facing conformation. Protein Sci 26: 130–145 27717063 10.1002/pro.3061 PMC5192975

[koaa020-B7] Curtis MD , Grossniklaus U (2003) A gateway cloning vector set for high-throughput functional analysis of genes *in Planta*. Plant Physiol 133: 462–469 14555774 10.1104/pp.103.027979 PMC523872

[koaa020-B8] Dubeaux G , Neveu J, Zelazny E, Vert G (2018) Metal sensing by the IRT1 transporter-receptor orchestrates its own degradation and plant metal nutrition. Mol Cell 69: 953–964 29547723 10.1016/j.molcel.2018.02.009

[koaa020-B9] Ellgaard L , Helenius A (2003) Quality control in the endoplasmic reticulum. Nat Rev Mol Cell Biol 4: 181–191 12612637 10.1038/nrm1052

[koaa020-B10] French AP , Mills S, Swarup R, Bennett MJ, Pridmore TP (2008) Colocalization of fluorescent markers in confocal microscope images of plant cells. Nat Protoc 3: 619–628 18388944 10.1038/nprot.2008.31

[koaa020-B11] Funakawa H , Miwa K (2015) Synthesis of borate cross-linked rhamnogalacturonan II. Front Plant Sci 6: 1–8 25954281 10.3389/fpls.2015.00223 PMC4404806

[koaa020-B12] Ghaddar K , Merhi A, Saliba E, Krammer E-M, Prévost M, André B (2014) Substrate-induced ubiquitylation and endocytosis of yeast amino acid permeases. Mol Cell Biol 34: 4447–4463 25266656 10.1128/MCB.00699-14 PMC4248734

[koaa020-B13] Gournas C , Saliba E, Krammer E-M, Barthelemy C, Prévost M, André B (2017) Transition of yeast Can1 transporter to the inward-facing state unveils an α-arrestin target sequence promoting its ubiquitylation and endocytosis. Mol Biol Cell 28: 2819–2832 28814503 10.1091/mbc.E17-02-0104 PMC5638585

[koaa020-B14] Guiney EL , Klecker T, Emr SD (2016) Identification of the endocytic sorting signal recognized by the Art1-Rsp5 ubiquitin ligase complex. Mol Biol Cell 27: 4043–4054 27798240 10.1091/mbc.E16-08-0570 PMC5156545

[koaa020-B15] Ho C-H , Lin S-H, Hu H-C, Tsay Y-F (2009) CHL1 functions as a nitrate sensor in plants. Cell 138: 1184–1194 19766570 10.1016/j.cell.2009.07.004

[koaa020-B16] Kasai K , Takano J, Miwa K, Toyoda A, Fujiwara T (2011) High boron-induced ubiquitination regulates vacuolar sorting of the BOR1 borate transporter in Arabidopsis thaliana. J Biol Chem 286: 6175–6183 21148314 10.1074/jbc.M110.184929 PMC3057829

[koaa020-B17] Keener JM , Babst M (2013) Quality control and substrate-dependent downregulation of the nutrient transporter Fur4. Traffic 14: 412–427 23305501 10.1111/tra.12039 PMC3594327

[koaa020-B18] Landi M , Margaritopoulou T, Papadakis IE, Araniti F (2019) Boron toxicity in higher plants: an update. Planta 250: 1011–1032 31236697 10.1007/s00425-019-03220-4

[koaa020-B19] Leitner J , Petrášek J, Tomanov K, Retzer K, Pařezová M, Korbei B, Bachmair A, Zažímalová E, Luschnig C (2012) Lysine63-linked ubiquitylation of PIN2 auxin carrier protein governs hormonally controlled adaptation of Arabidopsis root growth. Proc Natl Acad Sci USA 109: 8322–8227 22556266 10.1073/pnas.1200824109 PMC3361439

[koaa020-B20] Lin CH , MacGurn JA, Chu T, Stefan CJ, Emr SD (2008) Arrestin-related ubiquitin-ligase adaptors regulate endocytosis and protein turnover at the cell surface. Cell 135: 714–725 18976803 10.1016/j.cell.2008.09.025

[koaa020-B21] Lin WY , Huang TK, Chiou TJ (2013) NITROGEN LIMITATION ADAPTATION, a target of MicroRNA827, mediates degradation of plasma membrane-localized phosphate transporters to maintain phosphate homeostasis in Arabidopsis. Plant Cell 25: 4061–4074 24122828 10.1105/tpc.113.116012 PMC3877804

[koaa020-B22] Liu C , Shen W, Yang C, Zeng L, Gao C (2018) Knowns and unknowns of plasma membrane protein degradation in plants. Plant Sci 272: 55–61 29807606 10.1016/j.plantsci.2018.04.008

[koaa020-B23] Martins S , Dohmann EMN, Cayrel A, Johnson A, Fischer W, Pojer F, Satiat-Jeunemaître B, Jaillais Y, Chory J, Geldner N, et al (2015) Internalization and vacuolar targeting of the brassinosteroid hormone receptor BRI1 are regulated by ubiquitination. Nat Commun 6: 6151 25608221 10.1038/ncomms7151 PMC4713032

[koaa020-B24] Miwa K , Takano J, Omori H, Seki M, Shinozaki K, Fujiwara T (2007) Plants tolerant of high boron levels. Science 318: 1417–1417 18048682 10.1126/science.1146634

[koaa020-B25] Miwa K , Wakuta S, Takada S, Ide K, Takano J, Naito S, Omori H, Matsunaga T, Fujiwara T (2013) Roles of BOR2, a boron exporter, in cross linking of rhamnogalacturonan II and root elongation under boron limitation in Arabidopsis. Plant Physiol 163: 1699–709 24114060 10.1104/pp.113.225995 PMC3850200

[koaa020-B26] Moulinier-Anzola J , Schwihla M, De-Araújo L, Artner C, Jörg L, Konstantinova N, Luschnig C, Korbei B (2020) TOLs function as ubiquitin receptors in the early steps of the ESCRT pathway in higher plants. Mol Plant 13: 717–731 32087370 10.1016/j.molp.2020.02.012

[koaa020-B27] Nable RO (1988) Resistance to boron toxicity amongst several barley and wheat cultivars: a preliminary examination of the resistance mechanism. Plant Soil 112: 45–52

[koaa020-B28] Nable RO , Lance RCM, Cartwright B (1990) Uptake of boron and silicon by barley genotypes with differing susceptibilities to boron toxicity. Ann Bot 66: 83–90

[koaa020-B29] Pettersen EF , Goddard TD, Huang CC, Couch GS, Greenblatt DM, Meng EC, Ferrin TE (2004) UCSF Chimera – a visualization system for exploratory research and analysis. J Comput Chem 25: 1605–1612 15264254 10.1002/jcc.20084

[koaa020-B30] Reid RJ (2007) Identification of boron transporter genes likely to be responsible for tolerance to boron toxicity in wheat and barley. Plant Cell Physiol 48: 1673–1678 18003669 10.1093/pcp/pcm159

[koaa020-B31] Reid RJ , Hayes JE, Post A, Stangoulis JCR, Graham RD (2004) A critical analysis of the causes of boron toxicity in plants. Plant Cell Environ 27: 1405–1414

[koaa020-B32] Reithmeier RAF , Casey JR, Kalli AC, Sansom MSP, Alguel Y, Iwata S (2016) Band 3, the human red cell chloride/bicarbonate anion exchanger (AE1, SLC4A1), in a structural context. Biochim Biophys Acta Biomembr 1858: 1507–1532 10.1016/j.bbamem.2016.03.030 27058983

[koaa020-B33] Rodriguez-Furlan C , Minina EA, Hicks GR (2019) Remove, recycle, degrade: regulating plasma membrane protein accumulation. Plant Cell 31: 2833–2854 31628169 10.1105/tpc.19.00433 PMC6925004

[koaa020-B34] Schindelin J , Arganda-Carreras I, Frise E, **Kaynig V, Longair M, Pietzsch T, Preibisch S, Rueden C, Saalfeld S, Schmid B,** et al (2012) Fiji: an open-source platform for biological-image analysis. Nat Method 9: 676–682 10.1038/nmeth.2019 PMC3855844 22743772

[koaa020-B35] Sega GA (1984) A review of the genetic effects of ethyl methanesulfonate. Mutat Res Genet Toxicol 134: 113–142 10.1016/0165-1110(84)90007-1 6390190

[koaa020-B36] Sutton T , Baumann U, Hayes J, Collins NC, Shi B-J, Schnurbusch T, Hay A, Mayo G, Pallotta M, Tester M, et al (2007) Boron-toxicity tolerance in barley arising from efflux transporter amplification. Science 318: 1446–1449 18048688 10.1126/science.1146853

[koaa020-B37] Takano J (2006) The Arabidopsis major intrinsic protein NIP5;1 is essential for efficient boron uptake and plant development under boron limitation. Plant Cell 18: 1498–1509 16679457 10.1105/tpc.106.041640 PMC1475503

[koaa020-B38] Takano J , Kobayashi M, Noda Y, Fujiwara T (2007) Saccharomyces cerevisiae Bor1p is a boron exporter and a key determinant of boron tolerance. FEMS Microbiol Lett 267: 230–235 17166224 10.1111/j.1574-6968.2006.00556.x

[koaa020-B39] Takano J , Miwa K, Yuan L, von Wiren N, Fujiwara T (2005) Endocytosis and degradation of BOR1, a boron transporter of Arabidopsis thaliana, regulated by boron availability. Proc Natl Acad Sci USA 102: 12276–12281 16103374 10.1073/pnas.0502060102 PMC1189310

[koaa020-B40] Takano J , Noguchi K, Yasumori M, Kobayashi M, Gajdos Z, Miwa K, Hayashi H, Yoneyama T, Fujiwara T (2002) Arabidopsis boron transporter for xylem loading. Nature 420: 337–340 12447444 10.1038/nature01139

[koaa020-B41] Takano J , Tanaka M, Toyoda A, Miwa K, Kasai K, Fuji K, Onouchi H, Naito S, Fujiwara T (2010) Polar localization and degradation of Arabidopsis boron transporters through distinct trafficking pathways. Proc Natl Acad Sci USA 107: 5220–5225 20194745 10.1073/pnas.0910744107 PMC2841934

[koaa020-B42] Tamura K , Shimada T, Ono E, Tanaka Y, Nagatani A, Higashi S, Watanabe M, Nishimura M, Hara-Nishimura I (2003) Why green fluorescent fusion proteins have not been observed in the vacuoles of higher plants. Plant J 35: 545–555 12904216 10.1046/j.1365-313x.2003.01822.x

[koaa020-B43] Tanaka M , Sotta N, Yamazumi Y, Yamashita Y, Miwa K, Murota K, Chiba Y, Hirai MY, Akiyama T, Onouchi H, et al (2016) The minimum open reading frame, AUG-Stop, induces boron-dependent ribosome stalling and mRNA degradation. Plant Cell 28: 2830–2849 27760805 10.1105/tpc.16.00481 PMC5155345

[koaa020-B44] Tanaka M , Takano J, Chiba Y, Lombardo F, Ogasawara Y, Onouchi H, Naito S, Fujiwara T (2011) Boron-dependent degradation of NIP5;1 mRNA for acclimation to excess boron conditions in Arabidopsis. Plant Cell 23: 3547–3559 21908722 10.1105/tpc.111.088351 PMC3203445

[koaa020-B45] Thurtle-Schmidt BH , Stroud RM (2016) Structure of Bor1 supports an elevator transport mechanism for SLC4 anion exchangers. Proc Natl Acad Sci USA 113: 10542–10546 27601653 10.1073/pnas.1612603113 PMC5035872

[koaa020-B46] Vastermark A , Wollwage S, Houle ME, Rio R, Saier MH (2014) Expansion of the APC superfamily of secondary carriers. Proteins Struct Funct Bioinforma 82: 2797–2811 10.1002/prot.24643 PMC4177346 25043943

[koaa020-B47] Viotti C, Bubeck J, Stierhof Y-D, Krebs M, Langhans M, van den Berg W, van Dongen W, Richter S, Geldner N, Takano J, et al (2010) Endocytic and secretory traffic in *Arabidopsis* merge in the *trans*-Golgi network/early endosome, an independent and highly dynamic organelle. Plant Cell 22: 1344–1357 20435907 10.1105/tpc.109.072637 PMC2879741

[koaa020-B48] Wakuta S , Mineta K, Amano T, Toyoda A, Fujiwara T, Naito S, Takano J (2015) Evolutionary divergence of plant borate exporters and critical amino acid residues for the polar localization and boron-dependent vacuolar sorting of AtBOR1. Plant Cell Physiol 56: 852–862 25619824 10.1093/pcp/pcv011

[koaa020-B49] Wang S , Yoshinari A, Shimada T, Hara-Nishimura I, Mitani-Ueno N, Feng Ma J, Naito S, Takano J (2017) Polar localization of the NIP5;1 boric acid channel is maintained by endocytosis and facilitates boron transport in Arabidopsis roots. Plant Cell 29: 824–842 28341806 10.1105/tpc.16.00825 PMC5435427

[koaa020-B50] Yoshinari A , Fujimoto M, Ueda T, Inada N, Naito S, Takano J (2016) DRP1-dependent endocytosis is essential for polar localization and boron-induced degradation of the borate transporter BOR1 in *Arabidopsis thaliana*. Plant Cell Physiol 57: 1985–2000 27449211 10.1093/pcp/pcw121

[koaa020-B51] Yoshinari A , Hosokawa T, Amano T, Beier MP, Kunieda T, Shimada T, Hara-Nishimura I, Naito S, Takano J (2019) Polar localization of the borate exporter BOR1 requires AP2-dependent endocytosis. Plant Physiol 179: 1569–1580 30710051 10.1104/pp.18.01017 PMC6446798

[koaa020-B52] Yoshinari A , Korbei B, Takano J (2018) TOL proteins mediate vacuolar sorting of the borate transporter BOR1 in *Arabidopsis thaliana*. Soil Sci Plant Nutr 64: 598–605

[koaa020-B53] Yoshinari A , Takano J (2020) Analysis of endocytosis and intracellular trafficking of boric acid/borate transport proteins in Arabidopsis. Methods Mol Biol 2177: 1–13 32632800 10.1007/978-1-0716-0767-1_1

[koaa020-B54] Yoshinari A , Takano J (2017) Insights into the mechanisms underlying boron homeostasis in plants. Front Plant Sci 8: 1–8 29204148 10.3389/fpls.2017.01951 PMC5698777

