# Supplementary material for: Transport-coupled ubiquitination of the borate transporter BOR1 for its boron-dependent degradation
Source: Plant Cell. 2020 Dec 3;33(2):420–38. doi: 10.1093/plcell/koaa020 (PMC8136889; doi:10.1093/plcell/koaa020)
Supplement: koaa020_Supplementary_Data [file koaa020_supplementary_data.zip › tpc.00503.2020-s01.pdf]

Supplemental Data. Yoshinari et al. (2021). Plant Cell. Transport-Coupled Ubiquitination of the Borate Transporter BOR1 for Its Boron-Dependent Degradation.

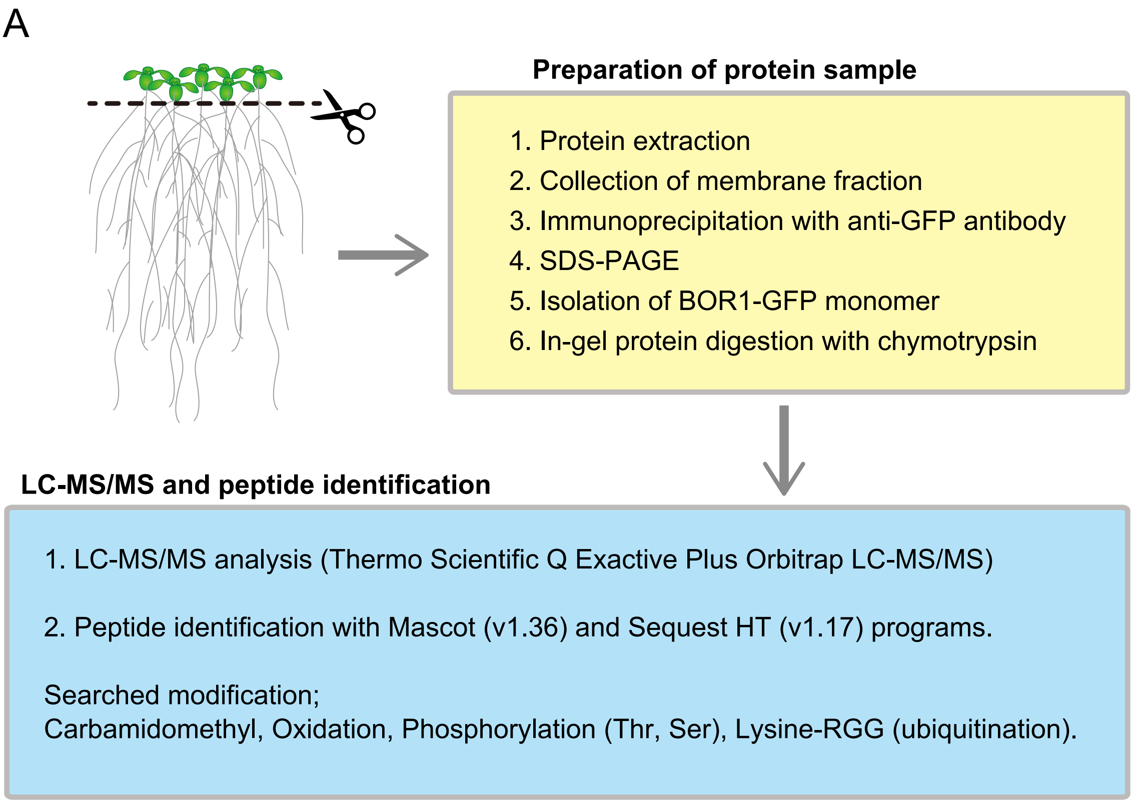

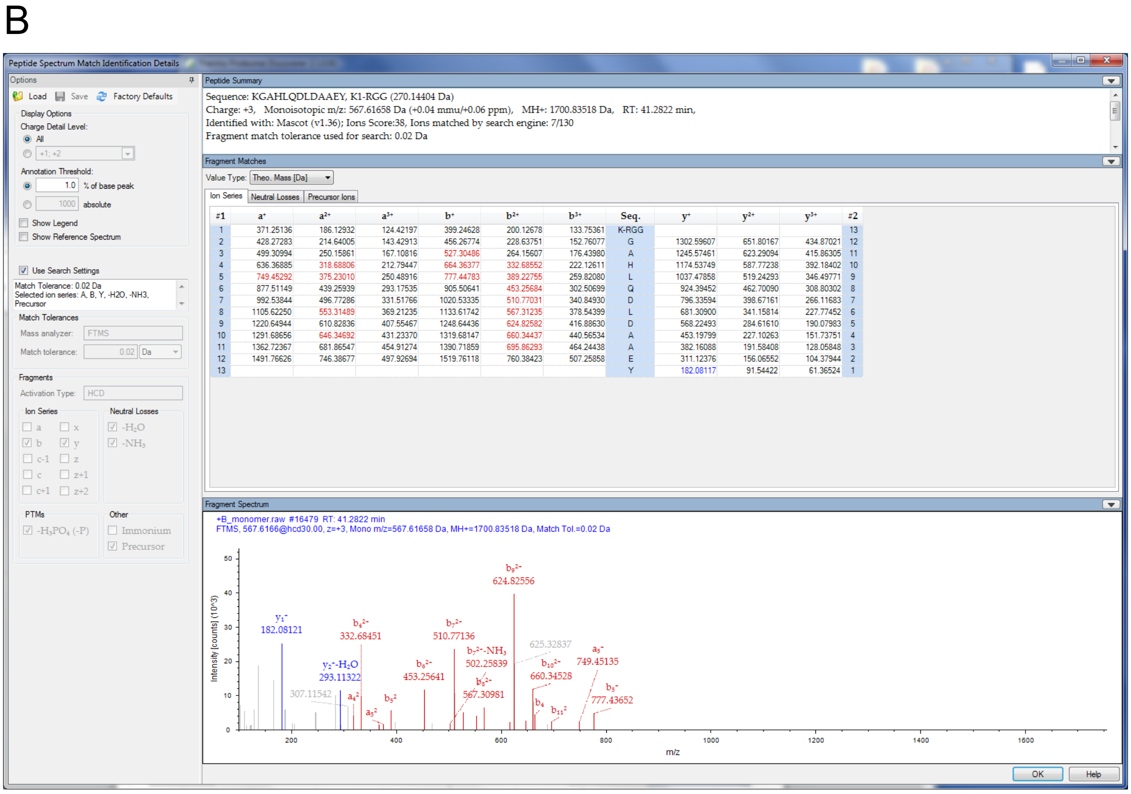

Supplemental Figure 1. LC-MS/MS Analysis (Supports Figure 1B). (A) Flow chart of LC-MS/MS analysis of BOR1-GFP. (1) BOR1-GFP was immunoprecipitated from Arabidopsis roots harboring *proBOR1:BOR1-GFP* by anti-GFP antibody. (2) Protein was separated by SDS-PAGE followed by isolation of BOR1-GFP monomer bands. (3) BOR1-GFP monomer was digested by chymotrypsin. (4) Peptides were subjected to LC-MS/MS and determined by Mascot (ver. 1.36) and Sequest HT (ver 1.17). **(B)** A representative result of identified peptides with RGG footprint at the K590 residue.

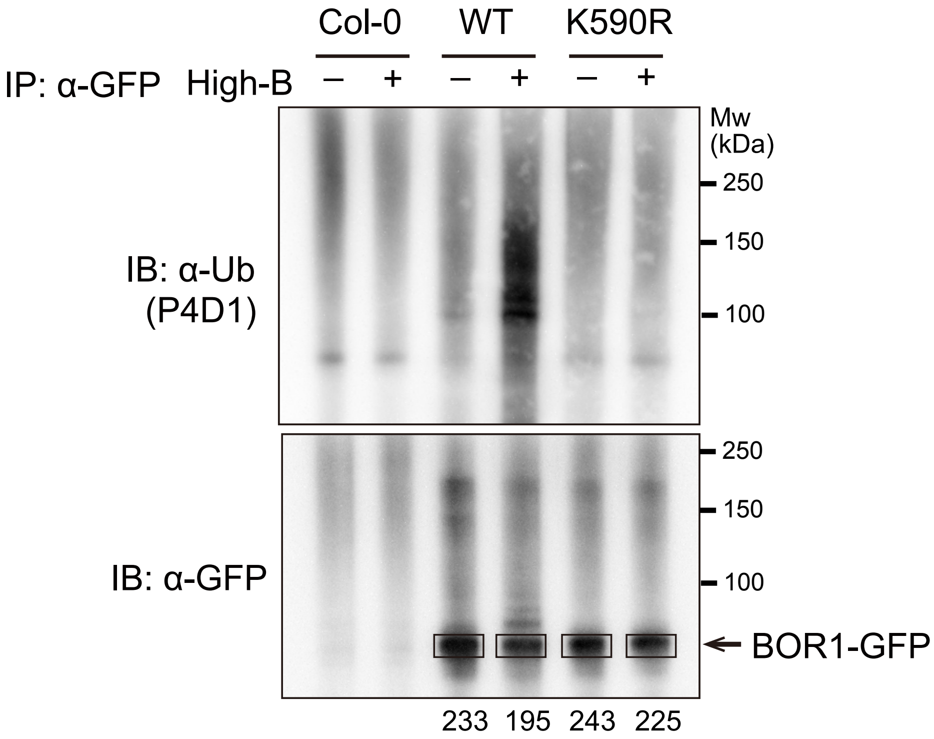

Supplemental Figure 2. Additional data of BOR1-GFP ubiquitination (Supports Figure 1C). Immunoblot analysis of immunoprecipitated BOR1-GFP treated with 0.5 (-) or 500 μM (+) B before protein extraction. Ubiquitin was detected by P4D1. Root-tissue lysates were used for immunoprecipitation. The values indicated below the image are signal intensities of the bands for unmodified BOR1-GFP selected by rectangular boxes. IP, immunoprecipitation; IB, immunoblot

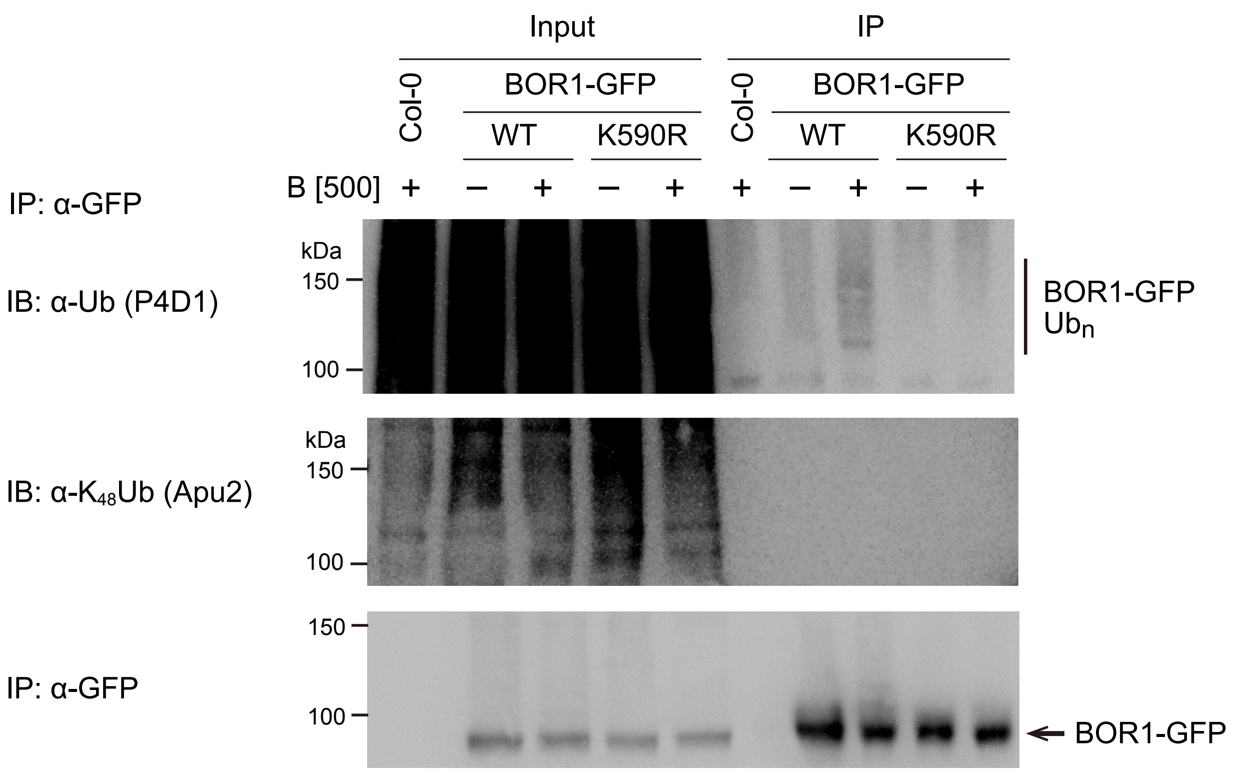

Supplemental Figure 3. K48-linked ubiquitination of BOR1-GFP was not detected (Supports Figure 1C). Immunoblot analysis of immunoprecipitated BOR1-GFP treated with 0.5 (-) or 500 μM (+) B before protein extraction. Ubiquitin and K48-linked ubiquitin chain were detected by P4D1 and Apu2 monoclonal antibodies, respectively. Root-tissue lysates were used for immunoprecipitation. Input represents lysates before immunoprecipitation. IP, immunoprecipitation; IB, immunoblot

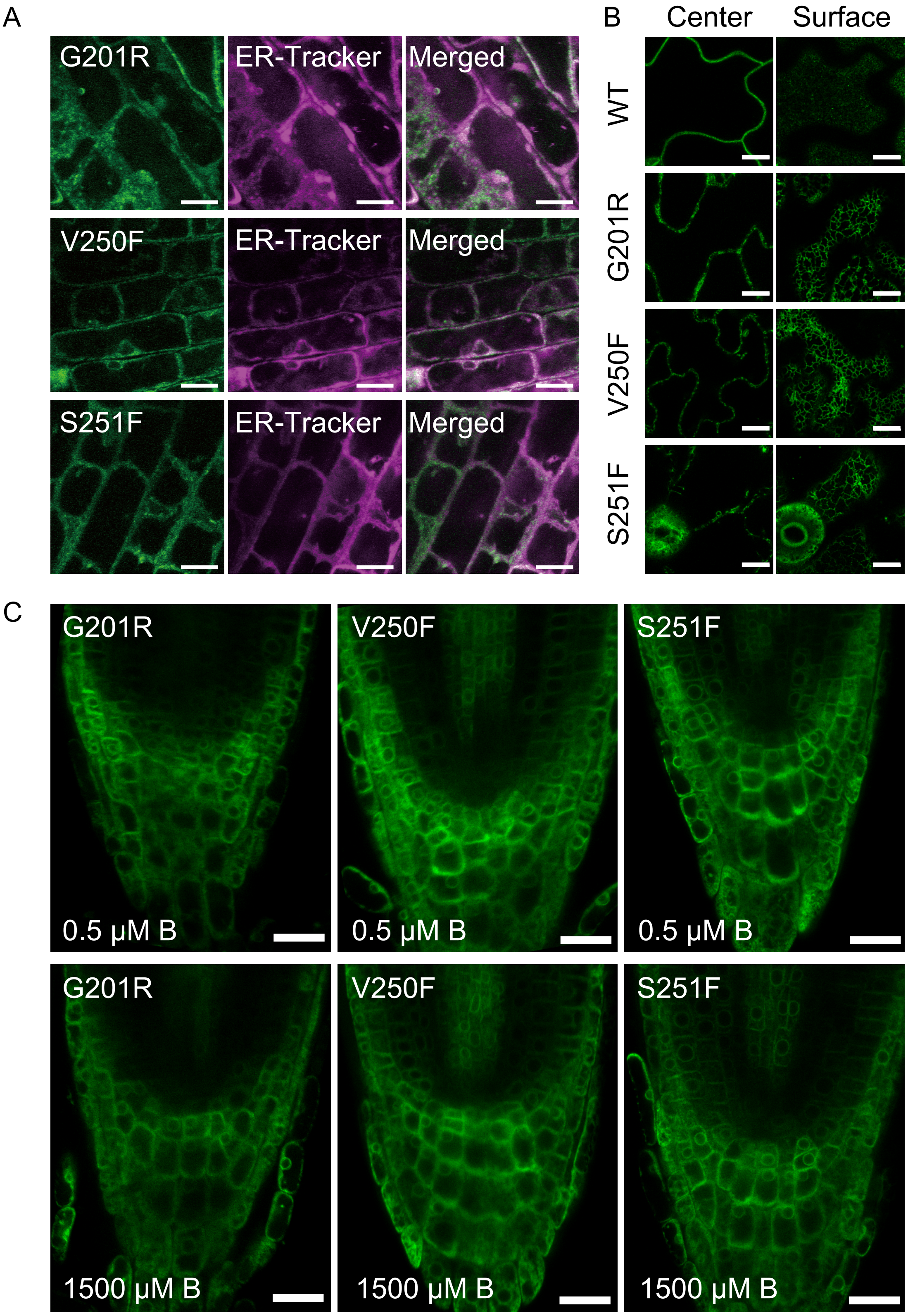

Supplemental Figure 4. Ectopic localization of G201R, V250F and S251F mutants of BOR1-GFP (Supports Figure 2). (A) ER-Tracker Red staining in the lateral root cap. BOR1-GFP was localized in the intracellular compartments stained by ER-Tracker Red. GFP signals were also found in punctate structures other than the ER. Plants were grown on a growth medium containing 0.5 µM B. **(B)** Central and surface optical sections of cotyledon epidermal cells expressing BOR1-GFP variants. The plasma membrane was labeled by WT BOR1-GFP while the network structures were labeled by the G201R, V250F and S251F mutants. **(C)** The intracellular localized BOR1-GFP variants do not show high B-induced degradation. Plants were grown on growth media containing 0.5 or 1500 µM B for 5 days. Scale bars represent 10 µm **(A, B)** and 20 µm **(C)**.

Supplemental Data. Yoshinari et al. (2021). Plant Cell. Transport-Coupled Ubiquitination of the Borate Transporter BOR1 for Its Boron-Dependent Degradation.

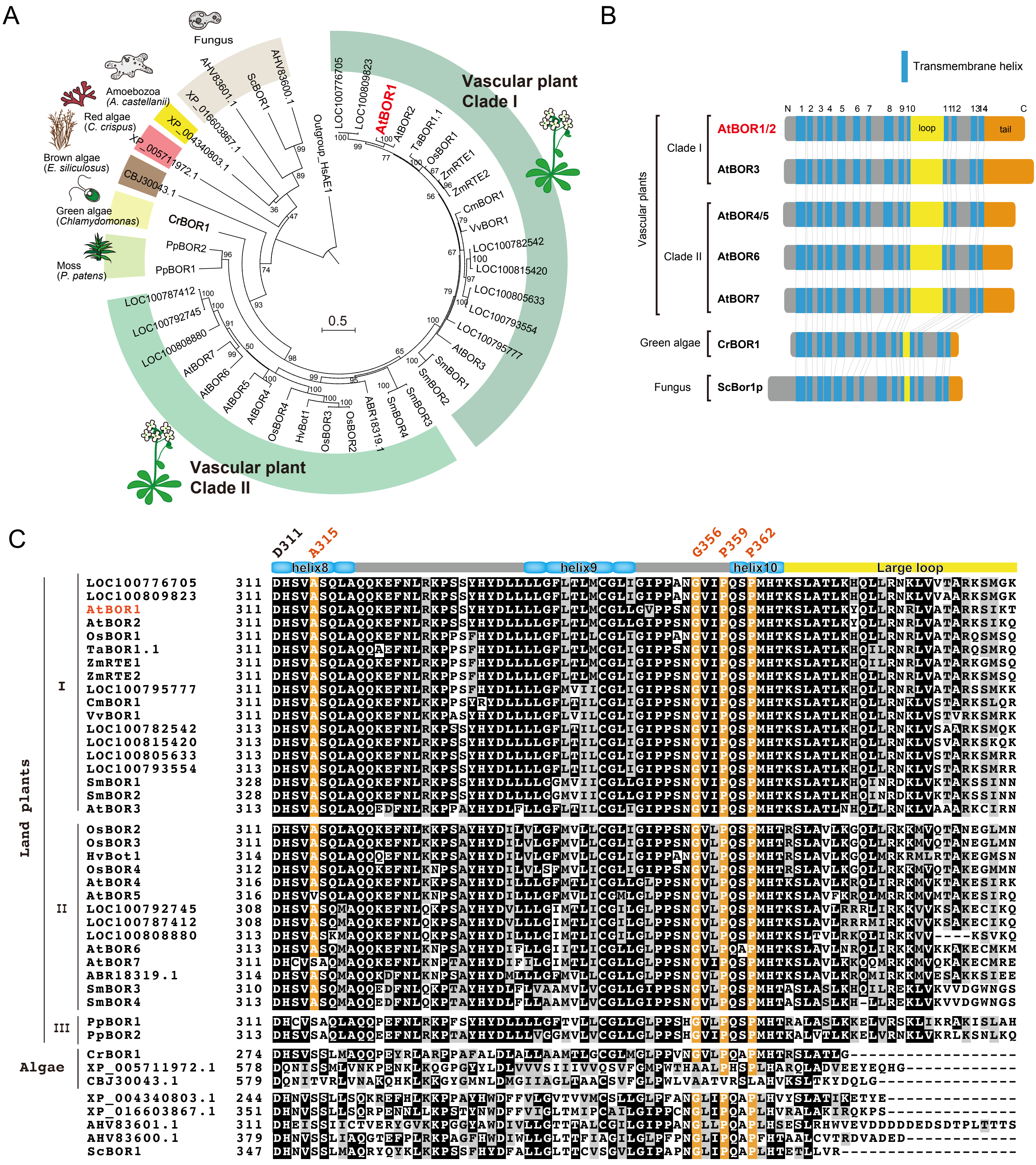

Supplemental Figure 5. Phylogenetic analysis of BOR family proteins (Supports Figure 3C). (A) A phylogenetic tree of BOR-type borate transporters and putative BOR homologues. **(B)** Structural comparison of *Arabidopsis thaliana* BORs, *Chlamydomonas reinhardtii* BOR1-like protein (CrBOR1), and *Saccharomyces cerevisiae* Bor1p. Cytosolic large loop and C-tail regions were highlighted in yellow and orange, respectively. **(C)** A multiple alignment of BORs and putative BOR family proteins. Accession

Supplemental Figure 5. Phylogenetic analysis of BOR family proteins (continued).

numbers: ScBOR1 (YNL275W), PpBOR1 (XP_024375985.1), PpBOR2 (XP_024398303), SmBOR1 (EFJ36311.1), SmBOR2 (EFJ09521.1), SmBOR3 (XP_002968573.1), SmBOR4 (BAR13154.1), AtBOR1 (AT2G47160), AtBOR2 (AT3G062270), AtBOR3 (AT3G06450), AtBOR4 (AT1G15460), AtBOR5 (AT1G74810), AtBOR6 (AT5G25430), AtBOR7 (AT4G32510), OsBOR1 (LOC_Os12g37840), OsBOR2 (LOC_Os01g08040), OsBOR3 (LOC_Os01g08020), OsBOR4 (LOC_Os05g08430), TaBOR1.1 (XP_020164523.1), ZmRTE1 (LOC100285382), ZmRTE2 (LOC100381433), CmBOR1 (ABQ52428.1), VvBOR1 (XP_002282501.1), HvBot1 (ABS83562.1), CrBOR1 (XP_001690501.1).

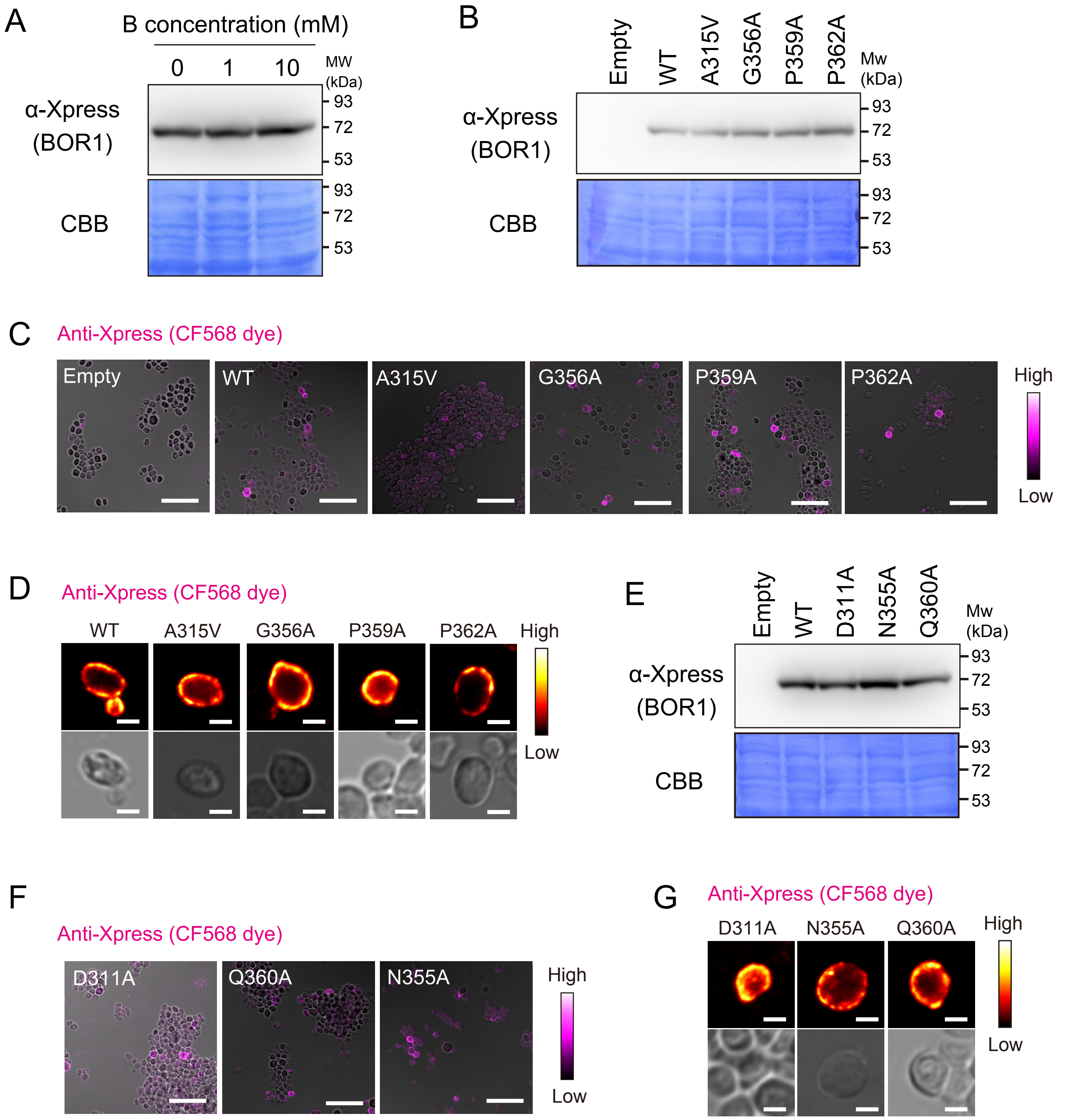

Supplemental Figure 6. Expression of Xpress-His6-BOR1 variants in yeast (Supports Figure 3D, 3E, 4B, and 4C). (A) Immunoblot analysis of Xpress-His6-AtBOR1 treated with different B concentration. Anti-Xpress antibody was used. CBB, coomassie brilliant blue. Yeast cells were incubated with SD-Ura+D-Galactose containing either 0, 1, or 10 mM boric acid for 1 h before protein extraction. **(B, E)** Detection of the Xpress-His6-AtBOR1 variants by immunoblot analysis. **(C, D, F, G)** immunofluorescence analysis of Xpress-His6-BOR1 variants in yeast. Scale bars represent 20 μm **(C, F)** and 2 μm **(D, G)**.

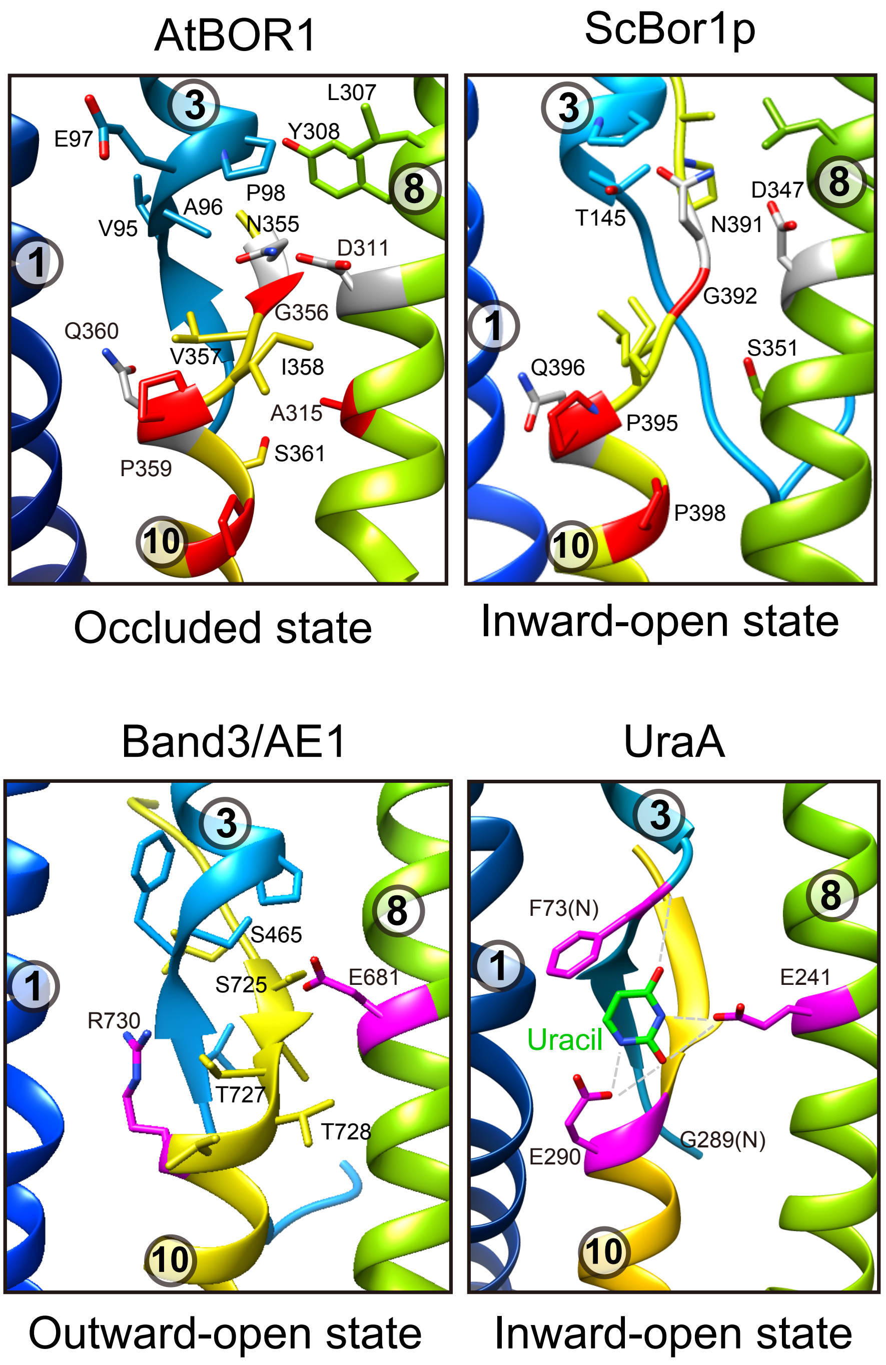

Supplemental Figure 7. Comparison of substrate-binding pockets of AtBOR1, ScBor1p, EcUraA, and HsBand3/AE1 (Supports Figure 4A). Structural data (AtBOR1, 5L25; ScBor1p, 5SV9; EcUraA, 3QE7; HsBand3/AE1, 4YZF) were obtained by PDB (https://www.rcsb.org/). Structural images were depicted by UCSF Chimera software ver. 1.13.1 (https://www.cgl.ucsf.edu/chimera/).

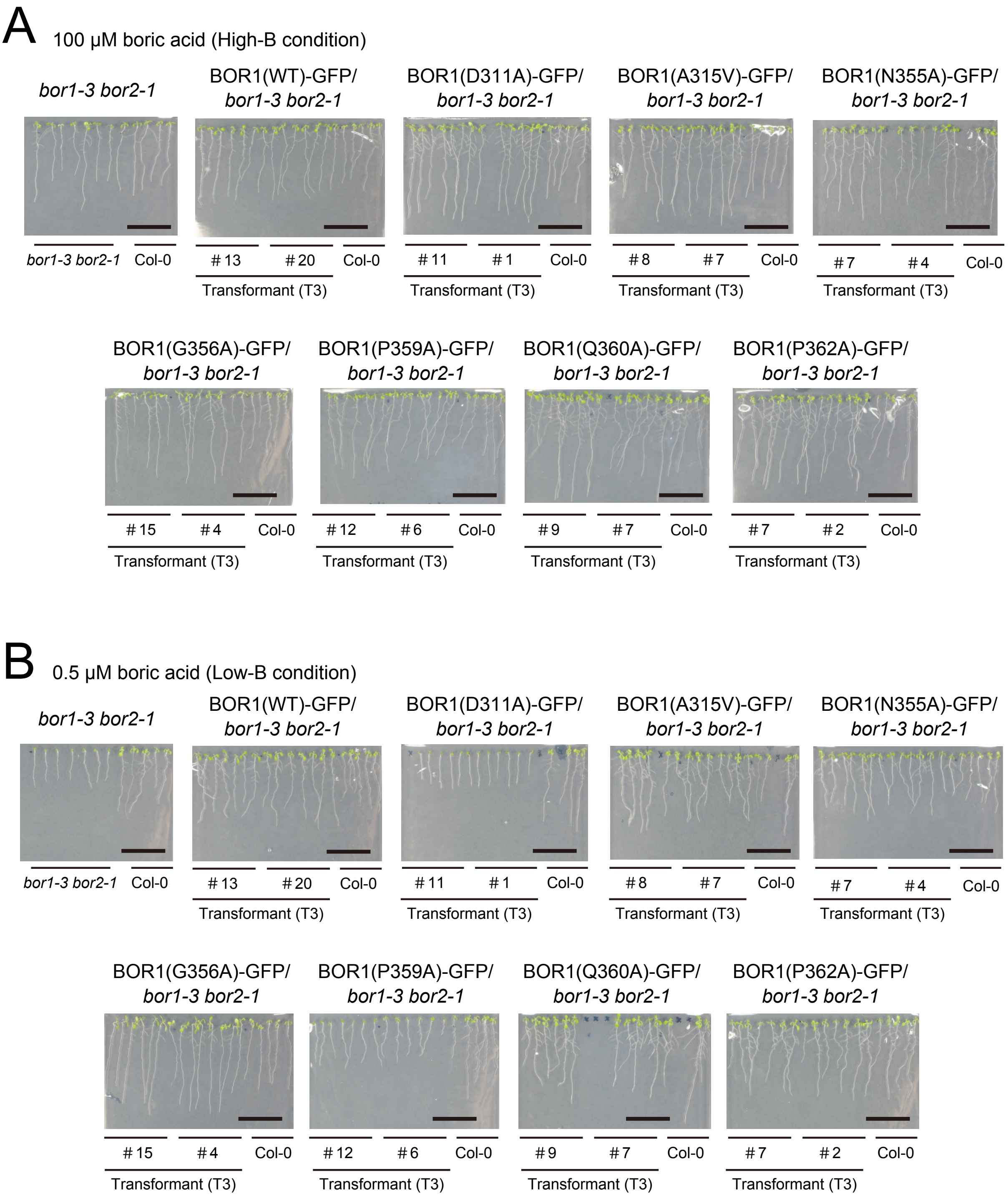

Supplemental Figure 8. Growth phenotype of transgenic plants expressing BOR1-GFP variants (Supports Figure 5D and 5E). (A, B) Growth phenotypes of 14-day-old seedlings of transgenic *bor1-3bor2-1* plants harboring *proBOR1:BOR1 variants-GFP* on MGRL media containing 100 μM (high-B) **(A)** and 0.5 μM (low-B) **(B)** boric acid. Two independent T3 lines were used for each construct. Scale bars indicate 3 cm.

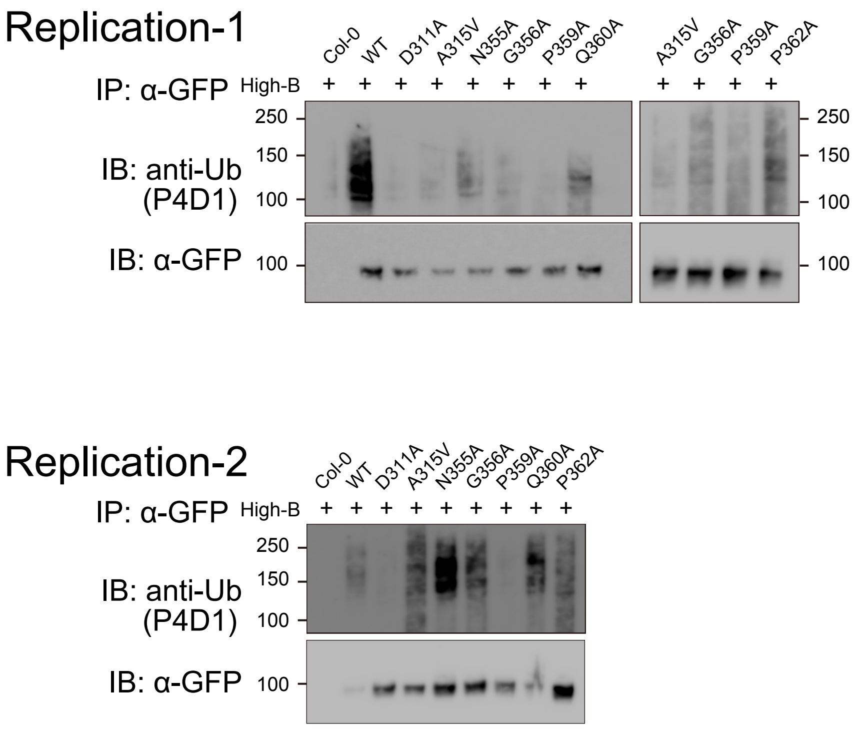

Supplemental Figure 9. Replication data of immunoblotting used for quantification of ubiquitination (Supports Figure 6A, 6B, and 6F). Relative ubiquitination levels of BOR1-GFP (Figure 6B) were calculated from three independent data sets, with 1 representative data set in Figure 6A, and the other 2 data sets in this figure.

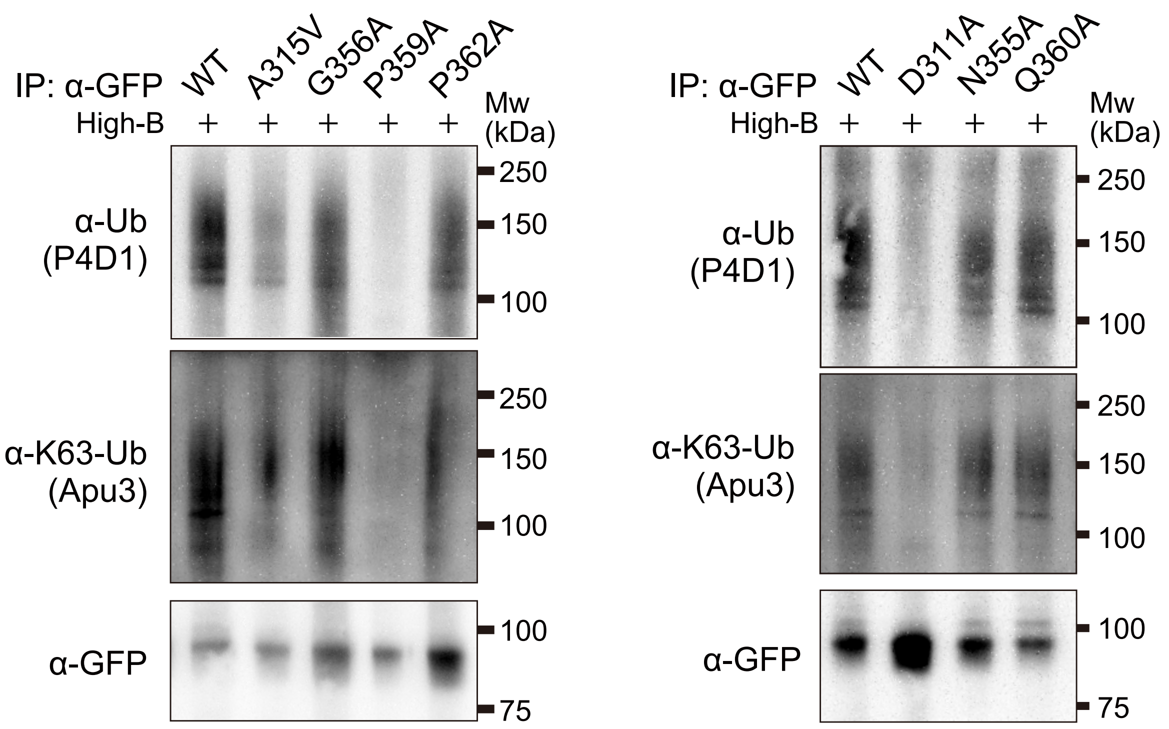

Supplemental Figure 10.K63-linked polyubiquitination is affected in BOR1-GFP variants (Supports Figure 6A). BOR1-GFP variants were immunoprecipitated from root-tissue-lysate of 14-day-old plants treated with 500 μM boric acid for 60 min. Ubiquitin and BOR1-GFP were detected by anti-ubiquitin monoclonal antibody (P4D1) and anti-GFP monoclonal antibody, respectively. Anti-ubiquitin mouse monoclonal antibody (P4D1), anti-GFP monoclonal antibody, and anti-K63-linked polyubiquitination antibody (Apu3) were used for immunoblotting.

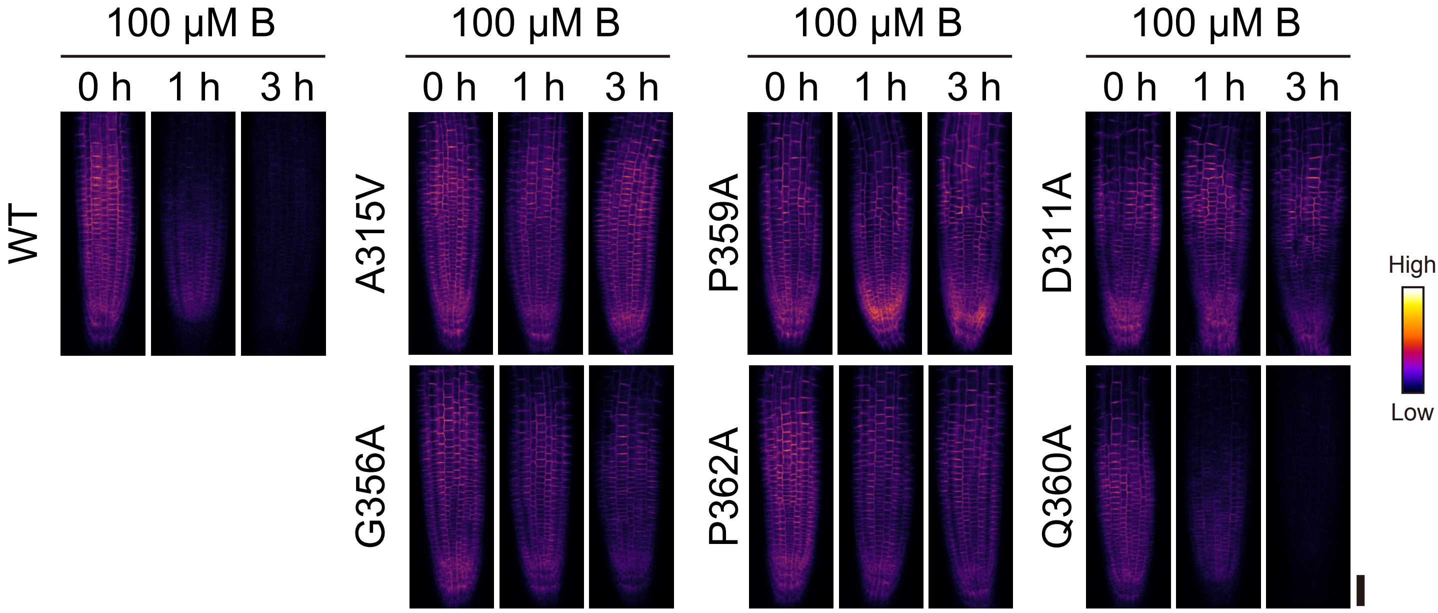

Supplemental Figure 11. Total fluorescence of BOR1-GFP variants in primary root tips (Supports Figure 6E). Plants were grown on MGRL media containing 0.5 μM B for 5 days followed by an incubation in high-B MGRL liquid medium (100 μM B) for 0, 1 or 3 h before imaging. A Z-stack comprised of 40 frames in 2 μm intervals over a total distance of 80 μm was taken by confocal microscopy. The images shown are the sum of all focal planes into one image by Z-project (sum slices) of Fiji/ImageJ software . The sum images were used for quantification shown in Figure 6E. Scale bar represents 50 μm.

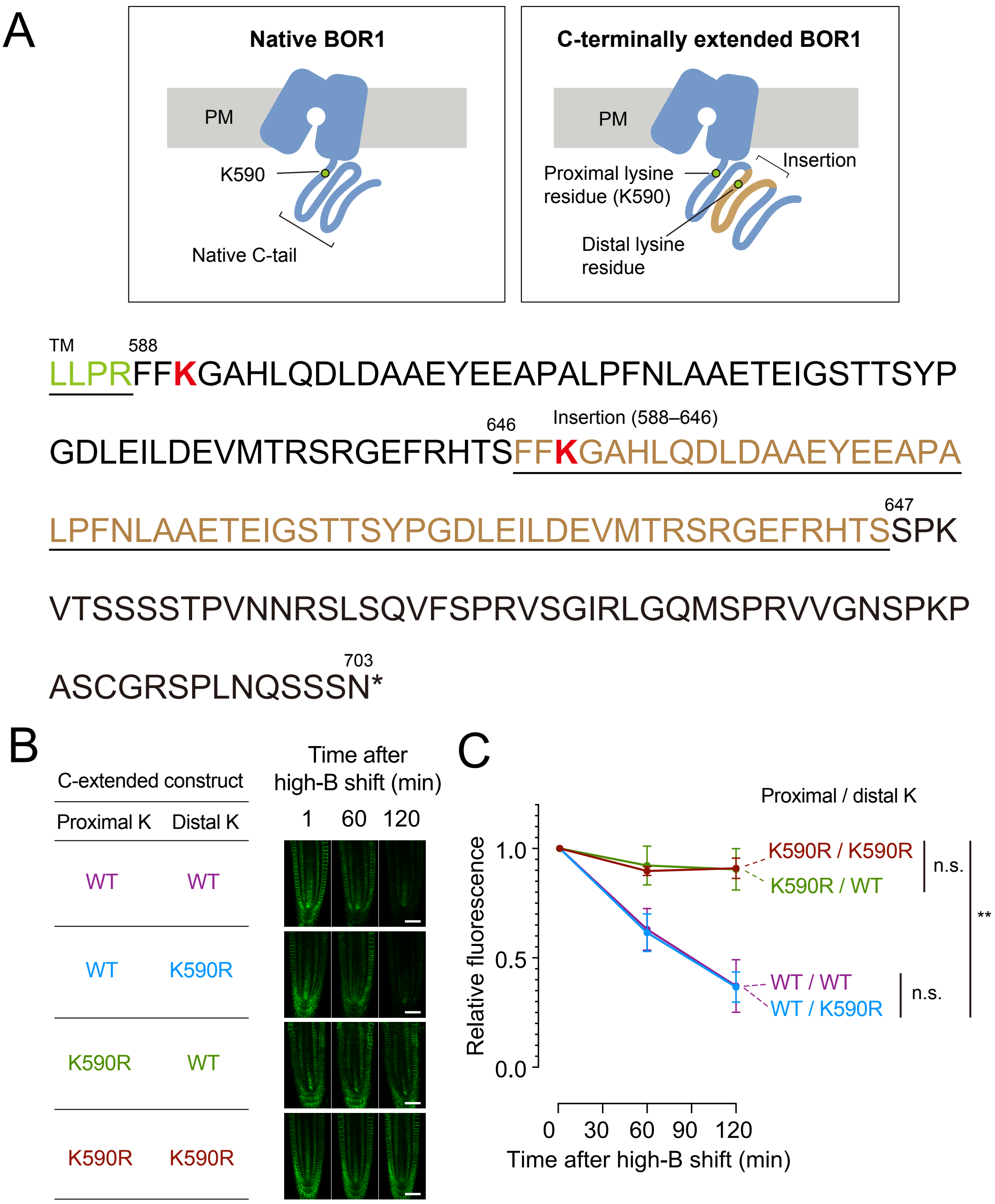

Supplemental Figure 12. C-terminal tail including K590 is not sufficient for B-induced degradation of BOR1 (Supports Figure 8). (A) Illustrations of wild-type and C-terminally extended BOR1. The 588th to 646th amino acid sequence was repeated in between the original 646th and 647th amino acid residues. Proximal K and distal K represent K590 in the native C-tail and K590 in the repeated C-tail sequence. The ubiquitination site (K590) was highlighted in red. PM, plasma membrane; TMD, transmembrane domain; *, carboxy terminus. (B) Confocal images of C-terminally extended BOR1-GFP at 1, 60, and 120 min after high-B (100 μM) supply. K/K, K/R, R/K, and R/R represent amino acid residues at proximal and distal K positions. Scale bars represent 50 μm. (C) Relative fluorescence of C-terminally extended BOR1-GFP constructs after high-B supply. A Z-stack comprised of 50 frames in 2 μm intervals over a total distance of 100 μm was taken for each individual root. Error bars represent mean ± SD. **P<0.01 by two-way ANOVA with Tukey-Kramer’ s post-hoc test. n.s. means no significant difference. *n* = 5 different roots.

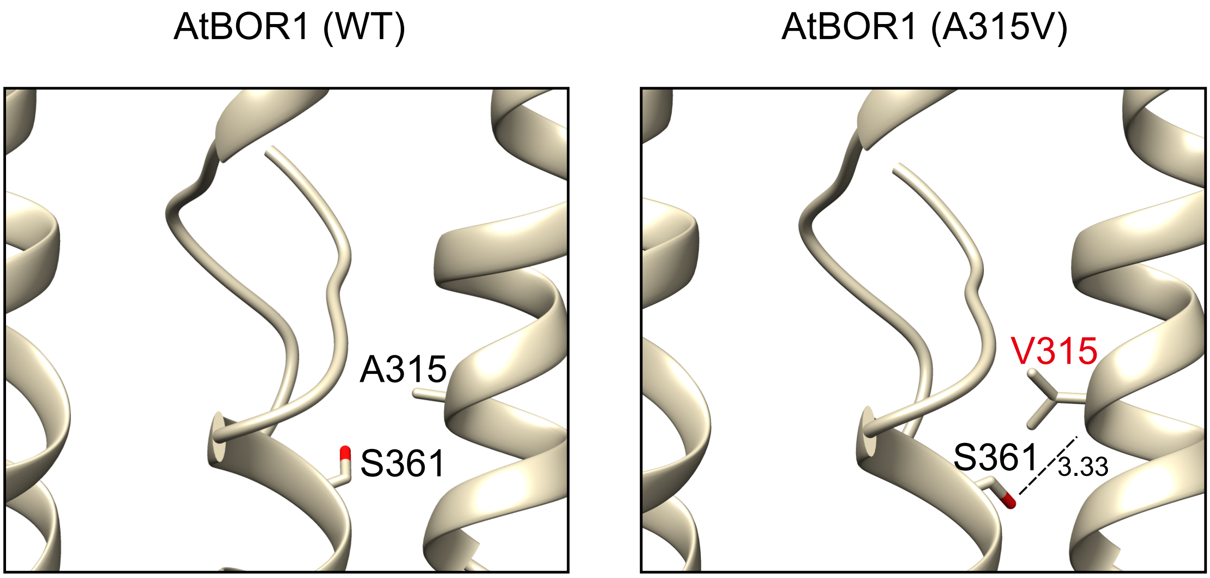

Supplemental Figure 13. *In-silico* prediction of impact of A315V substitution on BOR1 structure (Supports Figure 3A and 3B). The predicted structure around the substrate-binding pocket of WT (left) and A315V (right) substituted BOR1. An in-silico prediction indicates that the side chain of S361 and main chain of V315 possibly forms a hydrogen bond with a distance of 3.33 Å. The prediction was made with an online tool, Missense3D (http://www.sbg.bio.ic.ac.uk/~missense3d/)(Ittisoponpisan *et al.*, 2019).

Supplemental Table 1. Relative B-transport activity, poly-ubiquitination, and B-induced degradation rate of the BOR1-GFP variants.

	B-transport activity (^{11}B export in yeast)	Poly-ubiquitination[a] (Ub/GFP)	B-induced degradation[b] (Decrease within 3h)
WT	100	100	100
D311A	-6.1[c]	3	2
P359A	61	3	29
N355A	77	44	58
Q360A	91	61	93
A315V	98	22	-13[d]
G356A	95	18	35
P362A	102	18	32

[a] B-transport activity was determined as ^{11}B concentration in yeast with empty vector subtracted by that with each construct.
[b] B-induced degradation activity was determined as difference of BOR1-GFP fluorescence between 0 and 3 h after high-B supply.
[c,d] In the graphs of Figure 6F and 6G, 0 was used as the values.

Supplemental Table 2. Primer list

Name	Sequence (5'→3')	Purpose
K590R_For<br>K590R_Rev	CTTCAGAGGAGCTCATCTTCAGGACTTAG<br>CTCCTCTGAAGAATCTAGGGAGGAGATATTG	Introduction of K590R mutation
caccATGBOR1<br>sGFP stop	CACCATGGAAGAGACTTTTGTGCCGTTTG<br>TTACTTGTACAGCTCGTCCATGCC	Amplification of BOR1 sequence from Arabidopsis genomic DNA
BOR1_F1_FW<br>BOR1_F1_RV	GACGATGACGATAAGATGGAAGAGACTTTTGTGCC<br>GCCCTCTAGACTCGATCAGTTCGATGACGACTGG	Amplification of BOR1sequence for yeast expression vector
G356A_For<br>G356A_Rev	CAAACGCTGTCATTCCTCAATCTCCAATG<br>TGACAGCGTTTGATGGAGGGACTCCAAG	Introduction of G356A mutation
P359A_For<br>P359A_Rev	GTCATTGCTCAATCTCCAATGCATACCAAG<br>TTGAGCAATGACACCGTTTGATGGAGG	Introduction of P359A mutation
P362A_For<br>P362A_Rev	CTCAATCTGCAATGCATACCAAGAGCTTAGC<br>GCATTGCAGATTGAGGAATGACACCGTTTG	Introduction of P362A mutation
D311A_for<br>D311A_rev	ACTTCGCCCATAGTGTAGCTTCACAGCTC<br>ACTATGGGCGAAGTAGTAAAGCACAGCAATC	Introduction of D311A mutation
N355A_For<br>N355A_Rev	TCCATCAGGCGGTGTCATTCCTCAATC<br>ACACCGCCTGATGGAGGGACTCCAAG	Introduction of N355A mutation
Q360A_for<br>Q360A_rev	TTCCTGCATCTCCAATGCATACCAAGAG<br>TGGAGATGCAGGAATGACACCGTTTG	Introduction of Q360A mutation
Vector Forward Cter<br>Vector Reverse Cter<br>Vector Reverse 2 Cter	ACGAGTAGTCCTAAGGTGACAAGTTCAAG<br>TTTGAAGAAACTCGTGTGTCTAAACTCTCC<br>TCTGAAGAAACTCGTGTGTCTAAACTCTCC	Inverse PCR of Gateway entry vector of BOR1(WT/K590R)-GFP
Insert Forward Cter<br>Insert Forward 2 Cter<br>Insert Revers Cter	ACGAGTTTCTTCAAAGGAGCTCATC<br>ACGAGTTTCTTCAGAGGAGCTCATC<br>CTTAGGACTACTCGTGTGTCTAAACTCTCC	Amplification of C-terminal sequence with or without K590R mutation
