# Supplementary material for: Transport-coupled ubiquitination of the borate transporter BOR1 for its boron-dependent degradation
Source: Plant Cell. 2020 Dec 3;33(2):420–38. doi: 10.1093/plcell/koaa020 (PMC8136889; doi:10.1093/plcell/koaa020)
Supplement: koaa020_Supplementary_Data [file koaa020_supplementary_data.zip › tpc.00503.2020-s03.pdf]

((((((((((((LOC100776705:0.00858357,LOC100809823:0.01986593)1.0000:0
.09197723,
(AtBOR1:0.04523772,AtBOR2:0.04880617)1.0000:0.05653474)0.9970:0.0301
9565,(TaBOR1.1:0.05559438,(OsBOR1:0.03379442,
(ZmRTE1:0.02265794,ZmRTE2:0.01888508)0.9630:0.01054960)0.6730:0.0040
5370)1.0000:0.08530311)0.7770:0.01080820,
((CmBOR1:0.08516421,VvBOR1:0.08341190)0.7920:0.01067842,
((LOC100782542:0.00000000,LOC100815420:0.03512401)1.0000:0.05577151,
(LOC100805633:0.01501767,LOC100793554:0.01650095)1.0000:0.08502263)0
.9780:0.03118712)1.0000:0.05608645)0.5610:0.00914980,LOC100795777:0.
13611756)0.6760:0.01432792,AtBOR3:0.25987141)0.7920:0.01605009,
(SmBOR1:0.02865083,SmBOR2:0.00000000)1.0000:0.16342360)1.0000:0.0968
1552,((SmBOR3:0.01768359,SmBOR4:0.02008619)1.0000:0.35309238,
(ABR18319.1:0.18139905,
((((OsBOR2:0.01450374,OsBOR3:0.01149014)1.0000:0.06232663,HvBot1:0.1
0813349)1.0000:0.09441569,OsBOR4:0.18867169)1.0000:0.09917363,
((AtBOR4:0.05739321,AtBOR5:0.11765012)1.0000:0.16733776,
((AtBOR6:0.25084009,AtBOR7:0.23552355)0.9950:0.06126285,
(LOC100808880:0.17354494,
(LOC100792745:0.01405482,LOC100787412:0.01764090)1.0000:0.11101042)1
.0000:0.07089696)0.9180:0.04264691)0.5070:0.00686813)0.9980:0.095702
16)0.9560:0.08454260)0.4900:0.02089633)0.6580:0.04132507,
(PpBOR1:0.33986235,PpBOR2:0.19380348)0.9650:0.10285797)0.9820:0.2029
9382,XP_001690501.1:0.41014437)0.9320:0.27118506,CBJ30043.1:1.434184
63)0.7490:0.16715669,(XP_005711972.1:1.38276998,
((XP_016603867.1:0.80580340,XP_004340803.1:0.72653490)0.3610:0.04538
061,(AHV83601.1:1.25551024,
(ScBOR1:0.60604526,AHV83600.1:0.55395474)0.9910:0.35788394)0.8910:0.
22499481)0.8020:0.25247340)0.4760:0.04300131,Outgroup_HsAE1:1.535523
47);
