# Supplementary material for: Transport-coupled ubiquitination of the borate transporter BOR1 for its boron-dependent degradation
Source: Plant Cell. 2020 Dec 3;33(2):420–38. doi: 10.1093/plcell/koaa020 (PMC8136889; doi:10.1093/plcell/koaa020)
Supplement: koaa020_Supplementary_Data [file koaa020_supplementary_data.zip › tpc.00503.2020-s06.docx]

Dear Drs. Marcela Rojas-Pierce, Elizabeth Haswell, and Blake Meyers

Thank you very much for your kind handling and positive evaluation of our paper.

In the second revision process, we considered the comments made by Reviewer #3. The editorial comments were helpful. Regarding the concern about Fig.1C (K63-linked poly-ubiquitination detected by the Apu3 antibody), it is difficult for us to provide a better result within a reasonable time frame. Please see the response letter for more detail.

In the revision process, we carefully reviewed our results with the Apu3 antibody and realized a mistake in previous Supplemental Fig. 10 (K63-linked poly-ubiquitination of BOR1-GFP variants). The lower right panel (anti-GFP) was flipped horizontally. This mistake happened when we clipped and inverted images detected by different antibodies to prepare the figure. The correction (new Supplemental Fig.10) does not affect our conclusions and the degree of ubiquitination of BOR1-GFP variants based on the correct image indeed fits well to the quantification which was performed based on other images (Fig.6 and Supplemental Figure 9). We declare that this was simply a mistake and not intentional, and would like to apologize for our carelessness in the previous submissions.

We sincerely acknowledge the editors and reviewers for their effort and fair comments.

There are no new gene symbols in this manuscript.

We submitted supplemental data to support our article as follows:

Supplemental Figure 1. LC-MS/MS Analysis.

Supplemental Figure 2. Additional data of BOR1-GFP ubiquitination.

Supplemental Figure 3. K48-linked ubiquitination of BOR1-GFP was not detected.

Supplemental Figure 4. Ectopic localization of G201R, V250F and S251F mutants of BOR1-GFP.

Supplemental Figure 5. Phylogenetic analysis of BOR family proteins.

Supplemental Figure 6. Expression of Xpress-His6-BOR1 variants in yeast.

Supplemental Figure 7. Comparison of substrate-binding pockets of AtBOR1, UraA, and Band3/AE1.

Supplemental Figure 8. Growth phenotype of transgenic plants expressing BOR1-GFP variants.

Supplemental Fig. 9. Replication data of immunoblotting used for quantification of ubiquitination.

Supplemental Figure 10. K63-linked polyubiquitination is affected in BOR1-GFP variants.

Supplemental Figure 11. Total fluorescence of BOR1-GFP variants in the primary root tips.

Supplemental Figure 12. C-terminal tail including K590 is not sufficient for B-induced degradation of BOR1.

Supplemental Figure 13. *In-silico* prediction of the A315V substitution impact on BOR1 structure.

Supplemental Table 1. Relative B-transport activity, poly-ubiquitination, and B-induced degradation rate of the BOR1-GFP variants.

Supplemental Table 2. Primer list.

Author Revision Checklist.

I look forward to the comments on our manuscript.

Sincerely,

Junpei Takano

Graduate School of Life and Environmental Sciences, Osaka Prefecture University

1-1 Gakuen-cho, Naka-ku, Sakai, 599-8531, JAPAN

jtakano@plant.osakafu-u.ac.jp
