# Supplementary material for: Transport-coupled ubiquitination of the borate transporter BOR1 for its boron-dependent degradation
Source: Plant Cell. 2020 Dec 3;33(2):420–38. doi: 10.1093/plcell/koaa020 (PMC8136889; doi:10.1093/plcell/koaa020)
Supplement: koaa020_Supplementary_Data [file koaa020_supplementary_data.zip › tpc.00503.2020-s07.pdf]

Transport-coupled ubiquitination of a borate transporter BOR1 for boron-dependent degradation

Akira Yoshinari, Takuya Hosokawa, Marcel Pascal Beier, Keishi Ohshima, Yuka Ogino, Chiaki Hori, Taichi E. Takasuka, Yoichiro Fukao, Toru Fujiwara, Junpei Takano

Corresponding author: Junpei Takano jtakano@plant.osakafu-u.ac.jp

Review timeline:

TPC2020-RA-00503	Submission received:	June 30, 2020
	1st Decision:	July 29, 2020 *revision requested*
TPC2020-RA-00503R1	1st Revision received:	Oct. 2, 2020
	2nd Decision:	Oct. 26, 2020 *accept with minor revision*
TPC2021-RA-00503R2	2nd Revision received:	Oct. 30, 2020
	3rd Decision:	Oct. 30, 2020 *acceptance pending, sent to science editor*
	Final acceptance:	Nov. 20, 2020

REPORT: (The report shows the major requests for revision and author responses. Minor comments for revision and miscellaneous correspondence are not included. The original format may not be reflected in this compilation, but the reviewer comments and author responses are not edited, except to correct minor typographical or spelling errors that could be a source of ambiguity.)

TPC2020-RA-00503 1st Editorial decision – *revision requested* July 29, 2020

We have received reviews of your manuscript entitled "Transport-coupled ubiquitination of a borate transporter BOR1 for boron-dependent degradation." Thank you for submitting your best work to The Plant Cell. The editorial board agrees that the work you describe is substantive, falls within the scope of the journal, and may become acceptable for publication, pending revision and potential re-review.

We ask you to pay attention to the following points in preparing your revision:

- The immunoblot of Figure 6C has very unequal and overall weak signal in the anti-GFP panels such that comparisons between lanes is problematic. This result is critical to conclude different levels of ubiquitination between mutant forms of BOR1. Please provide a blot that shows more equal loading between samples. If access to the lab is restricted at this time, please consider providing this result from key mutants as validation.
- During post-review consultation, we agreed that it is not essential to provide evidence of conformational changes of BOR1 with and without B as suggested by reviewer 1, as we recognize that this would entail a significant amount of work beyond the timeline of a revision.
- Please clarify the interpretations of mutant phenotypes phenotypes as raised by Reviewer 2 and address all the issues raised by this reviewer.
- The ER tracker data does not fully support ER localization of intracellular mutants at this point. The staining with the dye in these cells does not show the typical network pattern of the ER and there are many blebs of the cytosolic signal. Co-localization with a fluorescent protein fusion should be included or simply describe this as "intracellular".

--- Reviewer comments:

Reviewer #1 (Comments for the Author):

In the manuscript "Transport-coupled ubiquitination of a borate transporter BOR1 for boron-dependent degradation" Yoshinari and coauthors fathom how the borate exporter BOR1 senses its substrate and how this leads to ubiquitination and subsequent degradation in the plant vacuole. In contrast to other plant transporters like NRT ot IRT sensing and transport capabilities are shown to be coupled and only the conformational change of BOR1 when binding borate allows ubiqutination by a (yet unknown) ligase.
The manuscript is exciting, statistics well conducted, the cited literature up to date and concept and organization of

experiments appealing. However, the proposed model that BOR1 functions as a transceptor is based on indirect evidence only.

Major concerns

To validate the proposed function of BOR1, protein confirmation studies could be conducted in presence and absence of B. Especially spectroscopical methods could be well suited here (e.g. CD spectroscopy, FTIR). I do understand that working with a relatively large membrane protein with 14 TMD is difficult but the fact that Thurtle-Schmidt and Strouda (2016) could purify BOR1 proves that it is possible. Successful identification of conformational change upon B-binding could confirm the proposed model and elevate the significance of the presented manuscript

Reviewer #2 (Comments for the Author):

The publication "Transport-coupled ubiquitination of a borate transporter BOR1 for boron-dependent degradation" Yoshinari et al. nicely and elegantly addresses the issue how the borate exporter BOR1 senses B and is consequently K63 poly-ubiquitylated at the K590 and further send to the vacuole for degradation. In an EMS screen, the authors identified amino acid residues essential for the degradation of BOR1 in response to elevated B levels, which are located close to the substrate-binding pocket of BOR1. Furthermore, mutations in other amino acids in the vicinity of the pocket were also assessed and they affected the B transport activity to various degrees. Several BOR1 variants, which lack B-transport activity showed reduced of poly-ubiquitylation and were therefore not degraded under higher B conditions. Thus, the authors of the study concluded that BOR1 functions as a B transceptor, because it senses the B concentration which promotes its own poly-ubiquitylation, which consequently leads to its vacuolar sorting. Understanding how downregulation of BOR1 is accomplished is of general interest for plant scientists and overall the manuscript is easy to read and the data are nicely presented. At this point, however, there are several points that should be clarified as listed below.

The authors start of this study with EMS mutants of BOR1-GFP that still have a plasma membrane localization but are stabilized under high B conditions: G356S, P359S and P362S. Is there an explanation why the A315V mutant is not mentioned in line 239, although it is also depicted in Figure 2D?

Furthermore, for to investigate the roles of these amino acid residues in B-transport, different mutants were used than the ones obtained in the EMS: G356A, P359A and P362A. Is there a reasoning behind this and if so, could the authors please comment on this.

In lines 281 the authors claim that the "P362 substitution had little effect on the B-export activity", (line 283) "the A315 did not show an obvious effect in the B-transport activity"...These two mutants behaved (according to Figure 3D and E) quite similarly though, which the authors also state I line 272/3 ("Introduction of the A315V and P362A variants enhanced the viability at levels comparable to WT, while G356A and P359A showed 274 apparently less viability (Figure 3D)"). Later on, the authors then differentiate between these two lines and claim that: line354... "P362A variants, which showed a slight reduction in the B-transport activity in yeast cells"... These differences are also depicted in Figure 6D and once again referred to in line 392 in the discussion. These differences need to be clarified and addressed more clearly as it is essential for the discussion of the results if these lines behave the same or differently.

This brings me to a general issue in the discussion that, although the data is very thorough and clearå and nicely presented, the model proposed in the discussion needs to be addressed more thoroughly. There are two mutant BOR1 lines A315V and P362A which are not impaired in B-transport, but are not degraded in high B-treatment. On the other hand, Q360A but also N355A show defects in B-transport, but are still ubiquitylated (although not as efficiently) and are degraded in high B conditions. How can these results be reconciled with the proposed model?

Reviewer #3 (Comments for the Author):

In the submitted paper, the authors suggest an interesting mechanism for BOR1 regulation coupling the borate transport and the protein degradation. They use a forward genetic screening in order to determine important residues for BOR1 stability. The obtained results show the involvement of the substrate-binding pocket; therefore, they also

characterize residues expected to be involved in B-transport . GFP-BOR1 mutant forms in these specific residues are expressed in yeast and in Arabidopsis in order to pin down their role. D311 is shown to be essential for B transport as well as for the protein degradation. Other residues, instead, are shown to have a lower impact. This is interesting; nevertheless, there are a few concerns, which need to find answers before considering publication.

Major comments:

1. The novelty of figure 1 is the specific formation of k63 chains (even if BOR1 was already listed as a K63 ubiquitinated protein, for example in Johnson & Vert 2016). The MS analysis and the use of K590R mutant confirm that ubiquitination occurs directly on this residue, but is not very different compared to what was already reported by Kasai et al 2011. I suggest to phrase in a lighter way in the abstract and in the main text. Figure 1A Quantification of the high-B induced PM delocalization can add value to the data, even if it is a known mechanism. In figure 1C GFP-BOR1 K509R is less than BOR1-GFP and therefore it might be that the laddering is below the detection. Another explanation, since in figure S2 the amounts of these two proteins are more comparable, is an input or loading problem. To make the data stronger I suggest to load equal amount of these proteins (or more of the mutant as it was in Kasai 2011) and show input or ccb loading control.

2. After an EMS screening 7 missense mutations within BOR1 coding sequence are selected and divided in two groups. Group one affects intracellular localization and the second group affects the PM localization after high B treatment. In order to claim that group one mutations lead to ER accumulation quantification of the data will be necessary and the limited absence of co-localization between GFP-BOR1 and ER-tracker should be shown. I suggest also to include a cytosolic control since the proteins might also be accumulated there. Even if is not the main focus of the paper another way to show that is accumulated in the ER, might be check ERAD activity. In line 232 is said: "these mutations are considered to be independent of the ubiquitination of BOR1 protein". There is no proof that these proteins are not ubiquitinated. In line 514-515 ..."important residues for the plasma membrane targeting" I think is an overstatement it might be possible to check, in silico, if there is a PM targeting peptide that is affected, but it is more likely what stated in lines 516-517: ER exit is impaired. Please rephrase

3. Key experiments to link borate transport mutants to degradation are the one in figure 6. As a general comments in order to say that these proteins variants are transported to the vacuole (even if it is the most likely option), I suggest to include Concanamycin treatment, or phrase it as protein stability. For clarify the message I suggest to divide these results from the ones of figure 5 and 8: make a different paragraph. About the protein stability microscopy data figure 6A seedlings are treated with 0.5mM B while the quantification refers to 0.1mM (that is in supplements), it is a little confusing, might be more linear add quantification referring to 0,5mM treatment. Q360A 30' after treatment shows very bright dots/vesicles, if this is consistent it might be interesting check if this mutation has an impact on the trafficking after PM internalization. ConcA treatment and protein accumulation in a time course can be helpful also in this. Unfortunately, the immunoblots in figure 6C, especially the one on the left are blurry and the input or a loading control is missing. How many times have this been repeated? Compared to wt the laddering with anti ub of the mutants is less, but for many variants the GFP-protein detected is also much less dampening the message. Could the author examine if the laddering will still be less loading more protein? About the proteins degradation, assuming that loading is comparable, it might be helpful add quantification of the bands intensity. For example P362A GFP band intensity after B treatment is strongly reduced (it is also very close to the edge and hard to say if it is a complete lane), but in the summary degradation is reported as 2 arrows down. An other example comes from figure S9 (where there are no control conditions) the GFP band of P360 is still very strong (as much as D311A?) even if the protein should be degraded.

Line 414 if it is the distance from the membrane or the distance from another specific amino acid in the protein is something that is hard to say. Can, in silico, be predicted a change in k590 position during boron transport or in the D311A mutant? This model is very interesting, but with the reported experiment I think that is only possible say that the K should be in position 590 to be ubiquitinated and change in the conformation as well as membrane distance are speculative.

TPC2020-RA-00503R1 1st Revision received Oct. 2, 2020

Reviewer/editor comments and **author responses**:

We considered all comments and revised our manuscript as follows. Especially, multiple repetition of the ubiquitination analysis allowed us to quantify the rates of ubiquitination. This process significantly improved the quality of our manuscript and made the interpretation clear. We sincerely acknowledge the editors and reviewers for their effort and fair comments.

Editor comment: The immunoblot of Figure 6C has very unequal and overall weak signal in the anti-GFP panels such that comparisons between lanes is problematic. This result is critical to conclude different levels of ubiquitination between mutant forms of BOR1. Please provide a blot that shows more equal loading between samples. If access to the lab is restricted at this time, please consider providing this result from key mutants as validation.

We agree that this is a critical point. We have repeated the analysis with the complete set of mutants and tried to load equally. We have to admit that the best blot shown in the new Fig. 6A still does not show equal loading. However, the WT BOR1 lane showed relatively low abundance of the non-modified BOR1-GFP band and a higher abundance of ubiquitinated BOR1-GFP bands compared to the lanes with BOR1 variants. Moreover, we have quantified the rates of ubiquitination from three independent experiments (new Supplemental Figure 2) and clarified the different degrees of ubiquitination (new Fig. 6B). This progress enabled us to discuss the link between ubiquitination and B-transport activity more objectively.

Editor comment: During post-review consultation, we agreed that it is not essential to provide evidence of conformational changes of BOR1 with and without B as suggested by reviewer 1, as we recognize that this would entail a significant amount of work beyond the timeline of a revision.

Thank you very much for your decision and instruction. We will try hard to analyze the conformational change in the coming years.

Editor comment: The ER tracker data does not fully support ER localization of intracellular mutants at this point. The staining with the dye in these cells does not show the typical network pattern of the ER and there are many blebs of the cytosolic signal. Co-localization with a fluorescent protein fusion should be included or simply describe this as "intracellular".

We agree. To clarify the ER localization, we added images of epidermal cells in cotyledons (new Supplemental Figure 4B). BOR1-GFP G201R, V250F, and S251F variants showed the typical network pattern of the ER.

Reviewer #1:

In the manuscript "Transport-coupled ubiquitination of a borate transporter BOR1 for boron-dependent degradation" Yoshinari and coauthors fathom how the borate exporter BOR1 senses its substrate and how this leads to ubiquitination and subsequent degradation in the plant vacuole. In contrast to other plant transporters like NRT ot IRT sensing and transport capabilities are shown to be coupled and only the conformational change of BOR1 when binding borate allows ubiqutination by a (yet unknown) ligase.

The manuscript is exciting, statistics well conducted, the cited literature up to date and concept and organization of experiments appealing. However, the proposed model that BOR1 functions as a transceptor is based on indirect evidence only.

Thank you very much for the encouragement.

Major concerns

To validate the proposed function of BOR1, protein confirmation studies could be conducted in presence and absence of B. Especially spectroscopical methods could be well suited here (e.g. CD spectroscopy, FTIR). I do understand that working with a relatively large membrane protein with 14 TMD is difficult but the fact that Thurtle-

Schmidt and Stroud (2016) could purify BOR1 proves that it is possible. Successful identification of conformational change upon B-binding could confirm the proposed model and elevate the significance of the presented manuscript.

We acknowledge that the identification of a conformational change is a critical point for the validation of the model we propose. We will start and try hard to identify differential conformations after this paper. Analysis of the structure of the cytosolic regions of transporters is still a challenge. The cytosolic region of BOR1 was also not visualized by the previous structural analysis by Thurtle-Schmidt and Stroud (2016). The pioneering work on yeast amino acid transporters indicating a transport-coupled ubiquitination model was not based on structures of themselves but on a homology with bacterial amino acid transporters. We believe that our data with an analogy to those on yeast transporters provide reasonable evidence of the model and will lead to a definite proof in our future studies.

Reviewer #2:

The publication "Transport-coupled ubiquitination of a borate transporter BOR1 for boron-dependent degradation" Yoshinari et al. nicely and elegantly addresses the issue how the borate exporter BOR1 senses B and is consequently K63 poly-ubiquitylated at the K590 and further send to the vacuole for degradation. In an EMS screen, the authors identified amino acid residues essential for the degradation of BOR1 in response to elevated B levels, which are located close to the substrate-binding pocket of BOR1. Furthermore, mutations in other amino acids in the vicinity of the pocket were also assessed and they affected the B transport activity to various degrees. Several BOR1 variants, which lack B-transport activity showed reduced of poly-ubiquitylation and were therefore not degraded under higher B conditions. Thus, the authors of the study concluded that BOR1 functions as a B transceptor, because it senses the B concentration which promotes its own poly-ubiquitylation, which consequently leads to its vacuolar sorting. Understanding how downregulation of BOR1 is accomplished is of general interest for plant scientists and overall the manuscript is easy to read and the data are nicely presented. At this point, however, there are several points that should be clarified as listed below.

Thank you very much for your encouraging comments.

The authors start of this study with EMS mutants of BOR1-GFP that still have a plasma membrane localization but are stabilized under high B conditions: G356S, P359S and P362S. Is there an explanation why the A315V mutant is not mentioned in line 239, although it is also depicted in Figure 2D?

This was simply our mistake. We mention now the A315V mutant in new Line 199.

Furthermore, for to investigate the roles of these amino acid residues in B-transport, different mutants were used than the ones obtained in the EMS: G356A, P359A and P362A. Is there a reasoning behind this and if so, could the authors please comment on this.

Thank you very much. We agree that the explanation was not enough. To clarify, we added the following sentence. Revised Line 222: “In this and following experiments, we substituted G356, P359, and P362 to A to keep the non-polar nature of the amino acids, although the substitutions we identified in the screening were S substitutions.”

In lines 281 the authors claim that the "P362 substitution had little effect on the B-export activity", (line 283) "the A315 did not show an obvious effect in the B-transport activity"...These two mutants behaved (according to Figure 3D and E) quite similarly though, which the authors also state I line 272/3 ("Introduction of the A315V and P362A variants enhanced the viability at levels comparable to WT, while G356A and P359A showed apparently less viability (Figure 3D)"). Later on, the authors then differentiate between these two lines and claim that: line354... "P362A variants, which showed a slight reduction in the B-transport activity in yeast cells"... These differences are also depicted in Figure 6D and once again referred to in line 392 in the discussion. These differences need to be clarified and addressed more clearly as it is essential for the discussion of the results if these lines behave the same or differently.

This brings me to a general issue in the discussion that, although the data is very thorough and clear and nicely presented, the model proposed in the discussion needs to be addressed more thoroughly. There are two mutant

BOR1 lines A315V and P362A which are not impaired in B-transport, but are not degraded in high B-treatment. On the other hand, Q360A but also N355A show defects in B-transport, but are still ubiquitylated (although not as efficiently) and are degraded in high B conditions. How can these results be reconciled with the proposed model?

Thank you very much for this very important comment. We indeed differentiated the behaviors of A315V and P362A variants in different sentences inappropriately. In repeated yeast growth assays the P362A variant tended to show slightly reduced B-tolerance (Fig.3D). However, there was no significant change of B concentrations in yeast cells (Fig. 3E). In the revised manuscript, we categorized A315V, G356A, and P362A as transport-competent variants based on the statistical analysis of B concentrations in yeast cells. Although it is speculative, these mutations probably affect the protein conformation without affecting transport activity. To clarify the categorization into two mutant groups (transport-defective and transport-competent), we added correlation plots between the B-transport activity and degradation (new Figure 6G and Supplemental Table 1) instead of the summary table (original Figure 6F). The descriptions in the manuscript were revised.

<u>Reviewer #3:</u>

In the submitted paper, the authors suggest an interesting mechanism for BOR1 regulation coupling the borate transport and the protein degradation. They use a forward genetic screening in order to determine important residues for BOR1 stability. The obtained results show the involvement of the substrate-binding pocket; therefore, they also characterize residues expected to be involved in B-transport . GFP-BOR1 mutant forms in these specific residues are expressed in yeast and in Arabidopsis in order to pin down their role. D311 is shown to be essential for B transport as well as for the protein degradation. Other residues, instead, are shown to have a lower impact. This is interesting; nevertheless, there are a few concerns, which need to find answers before considering publication.

Thank you very much for your encouraging comments.

Major comments:

1. The novelty of figure 1 is the specific formation of k63 chains (even if BOR1 was already listed as a K63 ubiquitinated protein, for example in Johnson & Vert 2016). The MS analysis and the use of K590R mutant confirm that ubiquitination occurs directly on this residue, but is not very different compared to what was already reported by Kasai et al 2011. I suggest to phrase in a lighter way in the abstract and in the main text.

Thank you very much for the suggestions. In our understanding, BOR1 has not been identified as a K63 ubiquitinated protein. It is not described in Johnson & Vert (2016). However, we agree that our conclusion is mostly dependent on the finding in Kasai et al. 2011. In the revised manuscript we rephrased it in a lighter way.

Figure 1A Quantification of the high-B induced PM delocalization can add value to the data, even if it is a known mechanism.

We agree that quantitative data of high B-induced delocalization of BOR1-GFP is valuable. We have included a graph of the time-course analysis of BOR1-GFP fluorescence in the PM after high-B supply as new Fig. 1B.

In figure 1C GFP-BOR1 K509R is less than BOR1-GFP and therefore it might be that the laddering is below the detection. Another explanation, since in figure S2 the amounts of these two proteins are more comparable, is an input or loading problem. To make the data stronger I suggest to load equal amount of these proteins (or more of the mutant as it was in Kasai 2011) and show input or ccb loading control.

Thank you very much for this very important point. Regarding the loading amount, we have repeated the experiments in Fig. 1C. The conclusion (no laddering on K590R variant) is stable. However, the detection by Apu3 was somewhat difficult and the original data in Fig.1C are still the best. Therefore, we kept the images in Fig.1C and provided an additional result with equal amount of loading (but only with the general UB antibody) as new Supplemental Figure 2. Supplemental Figure 3 (previous Supplemental Figure 2) also

supports the conclusion. Therefore, we consider that the combination of Fig.1C, Supplemental Fig. 2, and Supplemental Fig. 3 denies the possibility that the laddering is below the detection limit.

2. After an EMS screening 7 missense mutations within BOR1 coding sequence are selected and divided in two groups. Group one affects intracellular localization and the second group affects the PM localization after high B treatment. In order to claim that group one mutations lead to ER accumulation quantification of the data will be necessary and the limited absence of co-localization between GFP-BOR1 and ER-tracker should be shown. I suggest also to include a cytosolic control since the proteins might also be accumulated there. Even if is not the main focus of the paper another way to show that is accumulated in the ER, might be check ERAD activity. In line 232 is said: "these mutations are considered to be independent of the ubiquitination of BOR1 protein". There is no proof that these proteins are not ubiquitinated. In line 514-515 ..."important residues for the plasma membrane targeting" I think is an overstatement it might be possible to check, in silico, if there is a PM targeting peptide that is affected, but it is more likely what stated in lines 516-517: ER exit is impaired. Please rephrase.

Thank you very much for the helpful comment. To clarify the accumulation in the ER, we analyzed the localization of BOR1-GFP WT, G201R, V205F, and S251F in cotyledon epidermis cells and provided the resulting data as new Supplemental Figure 4B. The results support that the WT is mainly in the PM, and the mutants are mostly in the network-like structure of the ER. We removed the sentence "these mutations are considered to be independent of the ubiquitination of BOR1 protein (Original Line 232) ". We also rephrased the following sentences.

> Revised Line 186 “In contrast to WT, the intracellular-type BOR1-GFP variants colocalized with intracellular compartments stained by ER-Tracker Red in the lateral root cap cells (Supplemental Figure 4A) and showed a network pattern in cotyledon epidermal cells (Supplemental Figure 4B).
>
> Original line 513 “Our genetic screen using BOR1-GFP identified G201, V250, and S251 residues located in the gate domain as important residues for the plasma membrane targeting (Supplemental Figure 3). These amino acid residues are probably essential for proper protein folding or structural stability of BOR1 for ER exit.”
>
> Revised Line 475 “Our genetic screen using BOR1-GFP identified G201, V250, and S251 residues located in the gate domain as important residues for ER exit (Supplemental Figure 4). These amino acid residues are probably essential for proper protein folding or structural stability of BOR1.”

3. Key experiments to link borate transport mutants to degradation are the one in figure 6. As a general comments in order to say that these proteins variants are transported to the vacuole (even if it is the most likely option), I suggest to include Concanamycin treatment, or phrase it as protein stability. For clarify the message I suggest to divide these results from the ones of figure 5 and 8: make a different paragraph.

Thank you very much. We analyzed the vacuolar trafficking of mutants under a dark condition and provided the result as new Fig. 6C. Under dark conditions, the degradation of GFP in the vacuole is slowed down (Tamura et al. 2003). This analysis clarified the different degrees of vacuolar trafficking and also showed that the degradation of certain mutants does not happen in an unconventional pathway.

We also changed the sequence of figures in Fig. 6 (ubiquitination -> degradation in the revised manuscript) and revised/added sentences and a reference (Tamura et al. 2003) in the Result section.

About the protein stability microscopy data figure 6A seedlings are treated with 0.5mM B while the quantification refers to 0.1mM (that is in supplements), it is a little confusing, might be more linear add quantification referring to 0,5mM treatment.

We agree. We have repeated experiments using 0.1 mM and replaced the representative images (new Fig. 6D). In the revised manuscript, experiments in Fig. 6 consistently use 0.1 mM B.

Q360A 30' after treatment shows very bright dots/vesicles, if this is consistent it might be interesting check if this mutation has an impact on the trafficking after PM internalization. ConcA treatment and protein accumulation in a time course can be helpful also in this.

It was indeed strong in the image. We have repeated the experiments and carefully analyzed our data. We concluded that it was not consistent. The tendency was also not observed in the new representative images (new Fig. 6D).

Unfortunately, the immunoblots in figure 6C, especially the one on the left are blurry and the input or a loading control is missing. How many times have this been repeated? Compared to wt the laddering with anti ub of the mutants is less, but for many variants the GFP-protein detected is also much less dampening the message. Could the author examine if the laddering will still be less loading more protein? About the proteins degradation, assuming that loading is comparable, it might be helpful add quantification of the bands intensity. For example P362A GFP band intensity after B treatment is strongly reduced (it is also very close to the edge and hard to say if it is a complete lane), but in the summary degradation is reported as 2 arrows down. Another example comes from figure S9 (where there are no control conditions) the GFP band of P360 is still very strong (as much as D311A?) even if the protein should be degraded.

We agree that this is a very important point. We have repeated the analysis multiple times and provided the best looking one in new Fig. 6A and the second and third in new Supplemental Figure 9. Although the loading of unmodified BOR1-GFP (detected by anti-GFP) is still variable among lanes, the WT BOR1 lane showed relatively low abundance of the non-modified BOR1-GFP band and a higher abundance of ubiquitinated BOR1-GFP bands compared to the lanes with BOR1 variants (new Fig. 6A). This indicates lower rates of ubiquitination on BOR1 mutants compared to the BOR1 WT. Furthermore, using the replicates, we quantified the rates of ubiquitination (new Fig. 6B). The quantification allowed us to analyze the relationship between B-transport activity and ubiquitination in a more objective way (new Fig. 6F).

<u>Additional changes following the Author Revision Checklist</u>

Statistics: In the time course analysis of BOR1-GFP degradation, we used Dunnett's post-hoc test to compare the values between WT and variants. In the revised manuscript, we changed the method to Tukey-Kramer's post-hoc test to compare the complete data set.

Phylogenetic analysis: We changed the program for multiple sequence alignment from Clustal W to MASCLE program. New phylogenetic tree was drawn by neighbor-joining method with an outgroup, Homo sapiens anion exchanger 1 (HsAE1).

Methods: We revised carefully to include all necessary information.

TPC2020-RA-00503R1 2nd Editorial decision – *accept with minor revision* Oct. 26, 2020

We have received reviews of your manuscript entitled "Transport-coupled ubiquitination of a borate transporter BOR1 for boron-dependent degradation." On the basis of the advice received, the board of reviewing editors would like to accept your manuscript for publication in The Plant Cell. This acceptance is contingent on a minor revision based on the comments of our reviewers, several of which could continue to improve the manuscript. In particular, please add a description of the methods used to calculate the polarity index.

<u>Reviewer #2:</u>

The authors have completely addressed my concerns with more data and/or clarifications. The current manuscript was greatly improved and fortified after following the comments of all the reviewers. I thank them for their efforts in addressing my points and I have no further comments.

<u>Reviewer #3:</u>

The revised version of "Transport-coupled ubiquitination of a borate transporter BOR1 for boron-dependent degradation" is significantly improved. The authors reorganized several figures, included quantifications and

complementary experiments making their data clearer. Most of the issues that were risen in the previous version have been solved.

I still have some concern about figure 1C. The main point of this blot is that BOR1 is K63-linked poly-ubiquitinated, however Apu3 antibody detection seems to be difficult. I think that a replicate including the result with Apu3 and equal loading is important (but I do not require this for the acceptance of the manuscript, only if the authors can manage to have this data, in a reasonable time).

Minor additional comments:

Figure 1C: please move IP anti GFP above High B, in this way it will be clearer that all the blots are IPs.

Line 173: I think, supplemental figure 4 here is not required.

Line 273: figure 4B and 4C.

Figure S10: please add in the picture that it is an IP.

Line 382: I suggest to change "B status" to B concentration or B availability.

Line 424: I suggest to say maintenance of B homeostasis propose BOR1 as a transceptor for the maintenance of nutrient homeostasis in general might be too strong, .

In the methods how the polarity index is calculated has not been described.

TPC2020-RA-00503R2 2nd Revision received	Oct. 30, 2020

Reviewer comments and **author responses**:

We considered all comments and revised our manuscript as follows. Especially, multiple repetition of the ubiquitination analysis allowed us to quantify the rates of ubiquitination. This process significantly improved the quality of our manuscript and made the interpretation clear. We sincerely acknowledge the editors and reviewers for their effort and fair comments.

Reviewer #2:

The authors have completely addressed my concerns with more data and/or clarifications. The current manuscript was greatly improved and fortified after following the comments of all the reviewers. I thank them for their efforts in addressing my points and I have no further comments.

Thank you very much.

Reviewer #3:

1. The revised version of "Transport-coupled ubiquitination of a borate transporter BOR1 for boron-dependent degradation" is significantly improved. The authors reorganized several figures, included quantifications and complementary experiments making their data clearer. Most of the issues that were risen in the previous version have been solved.

Thank you very much.

2. I still have some concern about figure 1C. The main point of this blot is that BOR1 is K63-linked poly-ubiquitinated, however Apu3 antibody detection seems to be difficult. I think that a replicate including the result with Apu3 and equal loading is important (but I do not require this for the acceptance of the manuscript, only if the authors can manage to have this data, in a reasonable time).

It is difficult for us to provide a better result within a reasonable time frame. Our other results showed a consistent pattern, although they are with a higher background. The result in Fig.1D is among the best in terms of equal loading and low background. Since the intensities of unmodified BOR1-GFP detected by anti-

GFP are comparable in lane 4 (WT +B) and lane 6 (K590R +B), we believe this result supports the conclusion that WT BOR1 but not the K590R variant undergoes K63-linked ubiquitination upon high-B supply.

Additional changes

1. In the revision process, we carefully reviewed our results with the Apu3 antibody and realized a mistake in previous Supplemental Fig. 10 (K63-linked poly-ubiquitination of BOR1-GFP variants). The lower right panel (anti-GFP) was flipped horizontally. This mistake happened when we clipped and inverted images detected by different antibodies to prepare the figure. The correction (new Supplemental Fig.10) does not affect our conclusions and the degree of ubiquitination of BOR1-GFP variants based on the correct image indeed fits well to the quantification which was performed based on other images (Fig.6 and Supplemental Figure 9). We declare that this was simply a mistake and not intentional, and would like to apologize for our carelessness in the previous submissions.

2. We corrected the sequence of Supplemental Figure 10 and 11 in the first page of Supplemental Figures and Line 772-773 in the main text.

TPC2020-RA-00503R2 3rd Editorial decision – *acceptance pending*	Oct. 30, 2020

We are pleased to inform you that your paper entitled "Transport-coupled ubiquitination of a borate transporter BOR1 for boron-dependent degradation" has been accepted for publication in The Plant Cell, pending a final minor editorial review by journal staff.

Final acceptance from Science Editor	Date
