# Supplementary figures and images for: Transport-coupled ubiquitination of the borate transporter BOR1 for its boron-dependent degradation
Source: Plant Cell. 2020 Dec 3;33(2):420–38. doi: 10.1093/plcell/koaa020 (PMC8136889; doi:10.1093/plcell/koaa020)

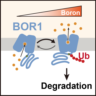

Supplement: koaa020_Supplementary_Data [file koaa020_supplementary_data.zip › tpc.00503.2020-s08.png]
